# Key Considerations for the Development of Safe and Effective SARS‐CoV‐2 Subunit Vaccine: A Peptide‐Based Vaccine Alternative

**DOI:** 10.1002/advs.202100985

**Published:** 2021-06-27

**Authors:** Ahmed O. Shalash, Waleed M. Hussein, Mariusz Skwarczynski, Istvan Toth

**Affiliations:** ^1^ School of Chemistry and Molecular Biosciences The University of Queensland St. Lucia QLD 4072 Australia; ^2^ Institute for Molecular Bioscience The University of Queensland St. Lucia QLD 4072 Australia; ^3^ School of Pharmacy The University of Queensland Woolloongabba QLD 4102 Australia

**Keywords:** angiotensin‐converting enzyme 2, antibody‐dependent enhancement, critical binding residues, neutralizing antibodies, receptor binding domain, SARS‐CoV‐2, spike protein, type‐I interferons

## Abstract

COVID‐19 is disastrous to global health and the economy. SARS‐CoV‐2 infection exhibits similar clinical symptoms and immunopathological sequelae to SARS‐CoV infection. Therefore, much of the developmental progress on SARS‐CoV vaccines can be utilized for the development of SARS‐CoV‐2 vaccines. Careful antigen selection during development is always of utmost importance for the production of effective vaccines that do not compromise recipient safety. This holds especially true for SARS‐CoV vaccines, as several immunopathological disorders are associated with the activity of structural and nonstructural proteins encoded in the virus's genetic material. Whole viral protein and RNA‐encoding full‐length proteins contain both protective and “dangerous” sequences, unless pathological fragments are deleted. In light of recent advances, peptide vaccines may present a very safe and effective alternative. Peptide vaccines can avoid immunopathological pro‐inflammatory sequences, focus immune responses on neutralizing immunogenic epitopes, avoid off‐target antigen loss, combine antigens with different protective roles or mechanisms, even from different viral proteins, and avoid mutant escape by employing highly conserved cryptic epitopes. In this review, an attempt is made to exploit the similarities between SARS‐CoV and SARS‐CoV‐2 in vaccine antigen screening, with particular attention to the pathological and immunogenic properties of SARS proteins.

## Graphical abstract

Careful antigen selection is essential for development of an effective and safe SARS‐CoV‐2 vaccine. Considering recent advances, peptide vaccines can be ideal substitutes for protein and RNA/DNA‐based vaccines. Peptide vaccines can avoid immunopathological sequences, focus immune responses on neutralizing B‐cell and on T‐cell epitopes, combine antigens from different viral proteins, and avoid mutant escape by employing conserved epitopes.

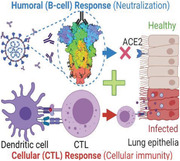

## 1 Introduction

SARS‐CoV‐2 (abbreviated here as SARS‐2) infection is the causative factor underlying coronavirus disease of 2019—more generally known as COVID‐19. The novel SARS‐2 outbreak, which initially presented in Wuhan in the Hubei province of China, rapidly transitioned into a worldwide pandemic, as declared by the WHO on March 11, 2020.^[^ ^1^ ^]^ In the 16 months since the first documented case, well over 160 million people (confirmed cases) have been infected with SARS‐2 and it has claimed over 3.3 million human lives. The overall mortality rate of the infection has been estimated to be around 2.1%.^[^ ^2^ ^]^ Currently (May 2021), the heaviest infection burden is in the United States, India, Brazil, France, Turkey, Russia, United Kingdom , Italy, Spain, Germany, Argentina, Columbia, Poland, Iran, Mexico, and Ukraine: each of these localities has more than 2 million infected cases. Positively, infections are mostly under control in some countries, including China, Canada, Australia, and Taiwan. However, it needs to be noticed that world infection burden is changing rapidly. The economic losses from the “lockdown” of cities and whole countries, the severely impacted tourism and transport industries, and the rapid shutdown of services provided by a diversity of small businesses across the globe have been estimated to be in the hundreds‐of‐billions of dollars.^[^ ^3^ ^]^

Three different zoonotic coronavirus epidemics have occurred in the past two decades. In addition to COVID‐19 disease, an outbreak of severe acute respiratory syndrome disease (SARS), which was caused by SARS‐1 virus, occurred in 2002 in the Guangdong province of China, and Middle East respiratory syndrome diseased, which was caused by MERS‐CoV virus, occurred in 2012 in Saudi Arabia (KSA). These viruses resulted in mortality rates of around 10% and 35%, respectively. The two previous outbreaks were well contained and, in each case, fatalities were limited to a few hundred.^[^ ^1^, ^4^, ^5^ ^]^ In contrast, containment efforts for SARS‐2 failed, and global spread ensued. However, the extensive research conducted on MERS‐CoV and SARS‐1 infections provided solid background for the development of treatments for SARS‐2 and facilitated rapid advancement of several vaccine candidates and antiviral drugs to clinical trials.^[^ ^6^, ^7^ ^]^ That being said, at present, there are still no approved therapeutic vaccines against any of the coronavirus infections: SARS‐1, MERS‐CoV, or SARS‐2. Recently, the DNA vaccine, nCoV‐19 ChAdOx‐1 (AstraZeneca/Oxford), and mRNA vaccines, mRNA‐1273 (Moderna), and BNT‐621 b1/b2 (Pfizer/Biontech), were approved for prophylactic human use. Several other vaccines, such as Ad26.COV2.S (Johnson & Johnson), Sputnik V (Gamaleya), and CoronaVac (Sinovac) have also been approved, but only for use in certain countries. Furthermore, the only approved antiviral medication (Remdesivir, Veklury) has very modest efficacy (15% survival compared to 11% for the placebo group, *n =* 521).^[^ ^8^ ^]^ Likewise, it failed to demonstrate significant benefits in treatment of moderate or severe illness in small clinical trials.^[^ ^9^, ^10^ ^]^

Notably, peptide‐based vaccines against these coronaviruses have been overlooked. However, they may hold great potential in providing safe and protective immune responses against SARS‐2 infections. Therefore, this review summarizes the immunogenicity and protective capacity of SARS antigens, as well as the pathological dangerous sequences mapped within the highly similar SARS proteome. In addition, adjuvant choice, animal models of infection, SARS vaccination approaches with relative efficacy, and potential adverse responses are discussed.

### 1.1 Genome of SARS‐2 Virus

SARS‐2 virus is a positive‐sense (+) ssRNA‐enveloped virus of the *Betacoronavirus* genera, Coronaviridae family. SARS‐2 (Accession no. MN908947.3) has a similar overall genome identity to several other coronaviruses, such as bat coronavirus RaTG13 (96% similar, Accession no. MN996532.1) and SARS‐CoV (SARS‐1) (82% similar, *Urbani* strain, Accession no. AY278741.1). RaTG13 has 99% genome coverage, while SARS‐1 has 88% coverage of the SARS‐2 genome. MERS‐CoV (Accession no. NC_019843.3), the causative agent of MERS coronavirus infections, appears to the most divergent of the group, with only 30% genome coverage of SARS‐2, as calculated using the Blast‐n server.^[^ ^11^ ^]^

**Figure 1:**
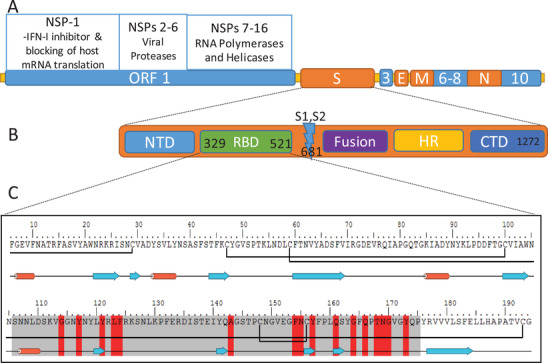
SARS‐2 genome, spike protein, and receptor binding domain (RBD). A) Viral RNA‐encoding structural (S, E, M, and N) and nonstructural proteins (NSPs). B) S‐protein subdomains. C) Sequence and structural conformation of RBD. In the RBD sequence: the residues highlighted in red are critical ACE2‐receptor binding residues; grey highlighted residues are the binding motif (RBM); the blue arrows indicate strand structure; the red cylinders indicate helical structure; and the black bonds between cysteine residues are native disulfide bonds.

Upon entry into host cells, SARS‐2 viral single‐stranded RNA transcription occurs after the translation of replicase–transcriptase enzymes from viral RNA. It encodes four structural proteins: spike (S), membrane (M), envelope (E) and nuclear (N) proteins, and 16 nonstructural proteins (NSPs) (**Figure** **1**). M‐protein plays a role in budding of the viral membrane and N‐protein is essential for the packaging of virus RNA.^[^ ^12^ ^]^ S‐protein plays a virulent role mediating viral attachment and fusion into host cells.^[^ ^13^, ^14^, ^15^ ^]^ E‐protein plays a key role in viral life cycle contributing to assembly and budding, and functions as ion‐channeling viroporin.^[^ ^16^ ^]^ The open reading frames (ORFs: portions of RNA sequence encoding amino acids without stop codons) encode several NSPs that play various roles in viral replication and the disruption of host immune responses. NSP‐1 inhibits host mRNA translation, while viral RNA helicases and polymerases, encoded in ORF1‐NSPs 7–16, transcribe viral RNA. Viral proteases, for example, PL^pro^ and 3CL^pro^, are encoded in ORF1‐NSPs 2–6, cleave the polyprotein precursor directly translated from viral RNA. Finally, in addition to NSPs, nine accessory factors are also encoded in viral RNA within several ORFs, that is, ORF 1, ORF 3, ORFs 6–9, and ORF 10, which interfere with host interferon production (Figure 1).^[^ ^17^ ^]^

### 1.2 Spike Protein and the Role of Receptor Binding Domain in Cell Entry

**Figure 2:**
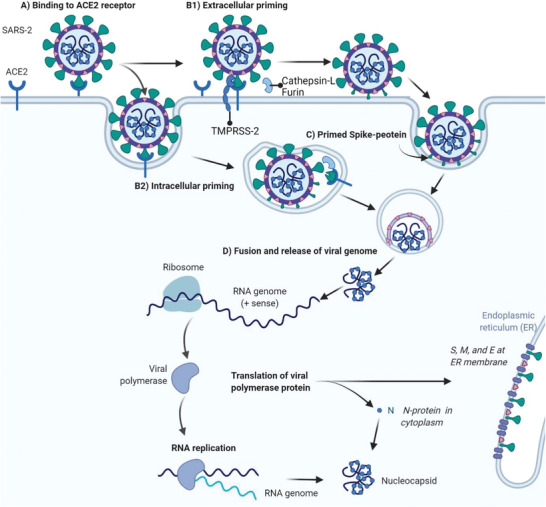
Schematic representation of A) binding, B) priming, C) S‐protein conformational changes, and the D) fusion process of SARS‐2 or SARS‐1 to a host cell bearing ACE2 receptor. A) SARS‐2 virus binds to the ACE2 receptor of a host cell via S‐protein RBD. B) Host proteases prime the S‐protein intra‐, or extracellularly. C) S‐protein adopts a hairpin coiled coils conformation and exposes the fusion peptide. D) Primed S‐protein with exposed fusion peptide drives virus fusion to the host cell, and viral RNA is injected for intracellular translation and transcription, and replication.

S‐protein is a homotrimeric transmembrane class‐I fusion glycoprotein that coats the surface of the SARS‐2 viral membrane. S‐protein is responsible for binding to the host cell surface receptor, fusing, and then entering into the host cell to initiate replication (**Figure** **2**).^[^ ^18^ ^]^ Therefore, S‐protein plays a key role in pathogenesis, virulence, and tissue invasion. SARS‐2 S‐protein (SARS‐2‐S) is homologous to SARS‐1 S‐protein (SARS‐1‐S) and RaTG13 S‐protein, with identity similarities of 75% and 98%, respectively, as calculated by the ClustalW server.^[^ ^19^ ^]^

Similar to other *Betacoronaviruses*, the SARS‐2‐S protein ectodomain has two main subunits and several domains: subunit 1 (S1) contains a receptor binding domain (RBD) located close to the S1/S2 cleavage site, while the S2 subunit contains fusion peptide (Figure 1B).^[^ ^13^, ^14^, ^15^, ^20^ ^]^ S‐protein trimer in the prefusion conformation is metastable and it can only bind to a receptor in its open conformation, that is, when one or more of the three RBDs are adopting the up‐position. The open, or “one‐up, two‐down” RBD conformation of S‐protein trimer was found to be most stable with 58% prevalence, while “two‐up” and “three‐up” conformations were less prevalent with 39% and 3%, respectively, in SARS‐1‐S. The one‐up, two‐down RBD conformation was also found to be most prevalent in SARS‐CoV‐2‐S.^[^ ^18^, ^21^ ^]^ SARS‐2‐RBD in up‐position binds to host cell angiotensin‐converting enzyme 2 (ACE2), which is expressed by lung type‐II pneumocytes and alveolar macrophages.^[^ ^13^, ^14^, ^15^, ^18^, ^22^, ^23^ ^]^ It is also worth noting that ACE2 is expressed in several other tissues, including muscles, adipose tissue, and the brain, kidney, and intestinal tract.^[^ ^24^ ^]^

The binding of SARS‐2‐S to host cell receptor ACE2 causes conformational changes that facilitate priming of the S‐protein (Figure 2A). S‐protein RBD binding to ACE2 reveals the S1/S2 cleavage site, and the host's proteolytic enzymes (e.g., furin, TMPRSS‐2, and cathepsin‐L) cleave at the polybasic (arginine‐rich) sites at the S1/S2 position, at ^681^RRAR^685^, and S2ʹ position, at KR^815^.^[^ ^21^, ^25^ ^]^ Cleavage by host enzymes sheds the S1 subunit, and exposes previously hidden S2 fusion machinery (Figure 2B,C).^[^ ^26^, ^27^ ^]^ The remaining S2 subunit irreversibly changes conformation into a coiled coils bundle of six helices with hairpin‐like structure that mediates fusion between the viral membrane and host cell membrane (Figure 2D).

**Figure 3:**
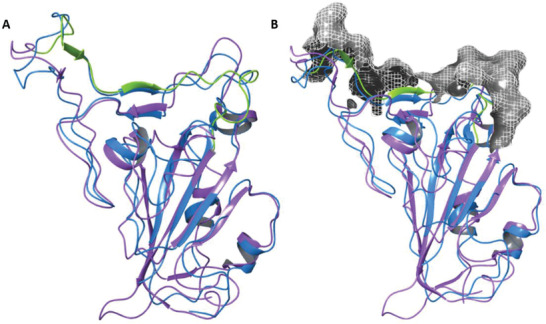
Comparison of SARS S‐proteins. A) Graphically aligned SARS‐2‐RBD (purple, from protein database, PDB: 6M0J) and SARS‐1‐RBD (blue, from PDB: 2AJF). SARS‐2‐RBM is marked in green. B) Graphically aligned RBDs of both viruses with a surface mesh electrostatic map of S‐protein RBM (grey) in contact with ACE2, produced using Schrodinger Bioluminate software. The close similarity between these two viral RBDs suggests the presence of common neutralizing epitopes.

The RBD of the SARS‐2 S‐protein (SARS‐2‐RBD) has 75% sequence similarity to SARS‐1‐RBD. However, the similarity between the receptor binding motifs (RBM: amino acid sequence within the RBD that is in intimate contact with the ACE2 receptor) is lower (59%) (Figure 1C).^[^ ^28^ ^]^ Albeit, several critical binding residues (CBRs) are conserved and some others are close analogous residues.^[^ ^22^ ^]^ The 6‐fold stronger binding affinity of SARS‐2‐RBD to ACE2 (≈15 nm) compared to SARS‐1‐RBD is naturally attributed to the amino acid sequence differences of the RBM region and also to minor, but significant, conformational changes due to modified disulfide bonds within the RBD (**Figure** **3**).^[^ ^18^, ^22^, ^29^ ^]^ Recently, it was reported that the exclusion of just one or two disulfide loops within the RBD of the S‐protein could completely prevent virus entry into cells.^[^ ^13^ ^]^

### 1.3 Host cell Receptor: Expression, Tissue Tropism, and Binding

ACE2 is a member of the dipeptidyl peptidase enzyme‐receptor family.^[^ ^30^ ^]^ It is an important part of the blood pressure regulation renin angiotensin system (RAS),^[^ ^31^ ^]^ as it cleaves angiotensin II (Ag‐II) and converts it into angiotensin (1–7), which reduces blood pressure. While SARS‐1 and SARS‐2 S‐proteins bind to ACE2 receptors to gain entry into a host cell, MERS‐CoV instead employs dipeptidyl peptidase‐4 (DPP‐4).^[^ ^30^ ^]^ ACE2 expression reflects viral host and tissue tropism. ACE2 is highly expressed in several human tissues, like lung, kidney. and heart tissue,^[^ ^32^, ^33^, ^34^, ^35^ ^]^ but has far lower expression in other tissues, for example, in the upper respiratory tract. This explains the very mild effects of SARS infections on the upper respiratory tract compared to the lower respiratory tract.^[^ ^36^, ^37^, ^38^ ^]^ The high ACE2 expression in type‐II pneumocytes in the lower respiratory tract and in heart ventricular tissues explains the severe illness sequelae, including elevated viral RNA load in the ventricles of deceased patients.^[^ ^38^, ^39^ ^]^ In the lungs, where the infection generally takes the strongest hold, SARS‐1 not only infects pneumocytes II, but also macrophages, as they too express ACE2.^[^ ^40^ ^]^ This causes damage to lung tissues as well as alveolar macrophages.

#### 1.3.1 Binding Characteristics of SARS‐2‐RBD to ACE2

The binding of both SARS viruses, through RBDs, occurs at the anionic ridge of ACE2 at the entrance to the catalytic site (position 636–717), but not within the catalytic site itself.^[^ ^20^, ^29^, ^41^, ^42^ ^]^ The RBD contains several amino acids that are able to form hydrogen bonds, and cationic amino acids that form electrostatic interactions or salt bridges with the anionic amino acids of the ACE2 ridge.^[^ ^29^ ^]^ The binding mode of RBDs to ACE2 is similar for both SARS viruses, and neither require glycosylation of the S‐protein. CBRs for SARS‐2‐RBD are K^417^, G^446^, Y^449^, Y^453^, L^455^, F^456^, F^486^, A^475^, N^487^, Y^489^, Q^493^, G^496^, Q^498^, T^500^, N^501^, G^502^, and Y^505^. These residues are located in the RBM region, position 438 to 506, in the S1‐subunit of the S‐ectodomain; they carry similar amino acids to SARS‐1.^[22,42]^ However, this does not mean that noncontact residues are unimportant; they impart the structural conformation necessary for the contact residues to bind effectively.^[^ ^20^, ^41^, ^42^ ^]^

#### 1.3.2 The Role of the Neuropilin‐1 Receptors in SARS‐CoV‐2 Host Cell Entry

Recently, SARS‐2 S‐protein was found to be able to bind to neuropilin‐1 (NRP‐1) receptors.^[^ ^43^ ^]^ The presence of this receptor affects tissue tropism in the presence of ACE2. The relative infectivity is 1.6‐fold higher with a combined presence of ACE2 and NRP‐1, compared to ACE2 alone in human embryonic kidney cells.^[^ ^13^, ^14^, ^43^ ^]^ The NRP‐1 receptor binds to the *C*‐terminus of RBD after S1/S2 cleavage by host enzymes. Therefore, S‐protein priming is critical for NRP‐1 to function as an entry receptor. Since priming occurs after conformational alteration to the S‐protein, which takes place due to ACE2 binding, NRP‐1 can enhance infectivity only in the presence of ACE2.^[^ ^43^ ^]^ NRP‐1 is expressed mainly in smooth muscles, lung cells, cardiac myocytes, and dendritic cells (DCs).^[^ ^44^ ^]^ It has been suggested that the presence of this alternative virus entry receptor, especially in the upper lung airways, could explain the more severe symptoms associated with SARS‐2 disease compared to SARS‐1 infections.^[^ ^43^ ^]^ However, if this were the case, then the increased tissue tropism and virulence would be dependent on the co‐expression of ACE2 and NRP‐1, as well as the priming enzymes, in the tissues. Since ACE2 is only highly co‐expressed with NRP‐1 in a few tissues, the overall effect would not be highly significant, that is, in the absence of ACE2. Still, it could be beneficial to include immunogenic epitopes near or at the cleavage site of S1/S2 in peptide vaccine to prevent NRP‐1 from binding to the *C*‐terminus of RBD after priming.

## 2 Immunopathology of Structural and Nonstructural SARS‐2 Proteins

The SARS‐1 proteome comprises several structural and NSPs that play immunopathological roles.^[^ ^45^, ^46^ ^]^ Unfortunately, no similar studies have been published on the immunopathology of sequences within SARS‐2 proteins so far. Coronavirus infections, especially SARS‐1 and SARS‐2,^[^ ^47^, ^48^ ^]^ are associated with the suppression of type‐I interferons (IFNs‐I) in patients showing severe illness (compared to those with mild illness)^[^ ^48^ ^]^ and the induction of pro‐inflammatory cytokine and chemokine production.^[^ ^49^, ^50^, ^51^, ^52^, ^53^ ^]^ IFNs‐I (IFN‐*α* and IFN‐*β*) can stimulate the immune system to generate appropriate virus‐specific adaptive immune responses that counter and locally contain infection within afflicted tissues. Inflammatory cytokines, on the other hand, can deteriorate patient clinical status rapidly and even more severely than viral replication itself. This is supported by the deterioration of SARS‐1‐infected patients’ clinical conditions, characterized with severe coagulopathy, coinciding with the increase in inflammatory cytokine level and infiltration of monocytes and neutrophils, rather than the level of viral load, that is, replication‐associated damage.^[^ ^54^, ^55^ ^]^ This highlights the essential importance of careful antigen and adjuvant selection in order to control the cytokine release profile and develop a safe adaptive immune response.

Since most of the influential immunopathological sequences in SARS‐1 viral proteins are similar (or identical) to those of SARS‐2, it is expected that they play the same role in infection. This is also supported by similar clinical symptoms and cytokine release profiles exhibited by both types of SARS infections (Figure S1, Supporting Information).^[^ ^49^, ^50^, ^51^, ^53^, ^56^ ^]^ SARS‐2 infection is characterized by leaky vasculature and infiltration of inflammatory neutrophils. In COVID‐19 severe illness, the spread of immunopathology radiates in the lower respiratory tract, resulting in fibrosis, diffuse alveolar damage, and pneumonia from the accumulation of cell debris, fluids and fibrils, thus compromising the gas exchange processes. The cytokine profile of SARS‐1‐infected patients is characterized by elevated expression of IL‐1ß, IL‐6, IL‐8, MCP‐1, and IP‐10 (CXCL‐10); this is similar to the elevated pro‐inflammatory cytokine expression of SARS‐2‐infected patients.^[^ ^51^, ^57^ ^]^ Elevated IL‐6 cytokine level, which is prominent in both infections, induces vascular permeability and the production of IL‐8, MCP‐1, and more IL‐6.^[^ ^58^ ^]^ In young patients, moderate levels of pro‐inflammatory cytokines and IFN‐*γ* have been reported; this could be a contributing factor to the comparatively low fatality rate of infected children.^[^ ^53^, ^56^, ^59^ ^]^

COVID‐19, like SARS disease, shifts the immune system toward an innate Th2 hyperreactivity response, while inhibiting Th1 responses by blocking IFN‐I signaling and production pathways. As a result, cytokine storm chemotaxis recruits inflammatory cell responses, which induce tissue‐damaging inflammatory immune responses, for example, IL‐8 recruits neutrophils, and CCL3 (MIP‐1*α*) signals a macrophage inflammatory response.^[^ ^55^ ^]^ These latter immune cells contribute to immunopathology, for example, neutrophil infiltration, as reported in severe illness symptoms and in deceased patients.^[^ ^49^, ^60^, ^61^, ^62^, ^63^ ^]^ Inflammatory cell recruitment results in acute damage to the lungs. Therefore, immunopathology‐causing sequences and viral components should be avoided for the production of safe vaccines.

Alveolar macrophages and respiratory dendritic cells are vital antigen presenting cells (APCs), which are normally responsible for pathogen sampling and phagocytosis, as well as the development of adaptive immune responses through the production of necessary Th1 or Th2 interleukins, or both. The infection of alveolar macrophages, albeit not well investigated to date, may play a key role in pro‐inflammatory cytokine storm release.^[^ ^45^ ^]^ Thus, activation of respiratory dendritic cells might be a more effective pathway to fight infection.^[^ ^64^ ^]^ Indeed, 100% of mice survived SARS‐1 (MA15) viral challenge following early depletion of alveolar macrophages.^[^ ^64^ ^]^

### 2.1 Inhibition of Type‐I Interferons and Signaling Pathways by SARS‐1 and SARS‐2

**Figure 4:**
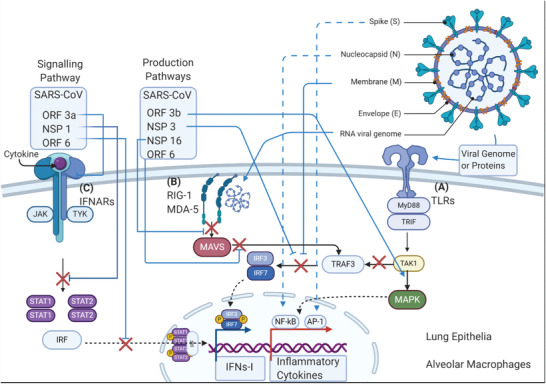
Schematic representation depicting IFN‐I production and signaling pathways. Pathways are depicted using black arrows between involved cofactors, adaptors, signal proteins and enzymes. Two pathways are involved in IFN‐I production: A) TLR‐TRAF3, and B) RIG‐1/MDA5‐MAVS, which are blocked by structural and nonstructural SARS‐1 proteins (blue lines and red x marks). C) A signaling pathway for IFN‐I production: IFNARs‐STAT1/2, which is blocked by nonstructural proteins ORF‐3a, ORF‐6, and NSP‐1. Simultaneously, inflammatory cytokine production pathways are stimulated through the TLR‐MAPK pathway (blue arrows) driving the translocation of NF‐*κ*B or AP‐1 via S‐protein, N‐protein, and ORF‐3b.

The production of IFNs‐1, such as IFN‐*α* and IFN‐*β*, is important in developing strong immune responses against viral infections. The pathways of IFN‐I production and signaling are blocked by SARS viral proteins.^[^ ^46^ ^]^ The production of IFNs‐I occurs through two main processes that start with either 1) the toll‐like receptor (TLR) pathway, or 2) the retinoic acid‐inducible gene 1 (RIG‐1) and melanoma differentiation‐associated protein 5 (MDA‐5) pathway (**Figure** **4**).^[^ ^65^, ^66^ ^]^ The TLR pathway starts with recognition of pathogen‐associated molecular patterns, including viral RNA or proteins, in the cytoplasm or on the cell surface (Figure 4, pathway A). RIG‐1 and MDA‐5 detect pathogenic patterns, mainly viral RNA in the cytoplasm (Figure 4, pathway B). Myeloid differentiation response‐88 (MyD88) and toll‐interleukin‐receptor (TIR)‐domain‐containing adapter‐inducing interferon‐*β* (TRIF) adaptor molecules are signal transduction adaptors of most TLRs. They initiate the transformational growth factor‐*β*‐activated kinase 1 (TAK‐1) cascade that activates nuclear transcription factors IRF‐3 and IRF‐7 by phosphorylation and translocation into the nucleus. This leads to the transcription of IFN‐I genes. In contrast, RIG‐1 and MDA‐5 employ adaptor protein mitochondrial antiviral signaling proteins (MAVs) that activate the complex formation of TNF receptor‐associated factor‐3 (TRAF‐3), along with several other factors and activators. These then phosphorylate IRF‐3 and IRF‐7, which results in their transport to the nucleus for IFN‐I transcription (Figure 4, pathway B).^[^ ^67^ ^]^

The major signaling pathways for IFN‐I (IFN‐*α* and IFN‐*β*) transcription genes occurs through interferon stimulating gene factor‐3 (ISGF‐3). This signaling pathway is initiated by the activation of Janus kinases (JAK‐1, TYK‐2) and signal transducers and activators of transcription (STAT‐1 and ‐2). Together, this is known as the JAK/STAT pathway (Figure 4, pathway C). Thereafter, the cell surface receptors (IFNAR) recognize IFNs, and JAK‐1 and TYK‐2 phosphorylate STAT‐1 and STAT‐2, which leads to the recruitment of transcription factor (IRF‐9) and the formation of the ISGF‐3 complex of IRF9, STAT‐1 and STAT‐2. The ISGF‐3 complex activates and amplifies the transcription of IFNs‐I.^[^ ^68^ ^]^

**Figure 5:**
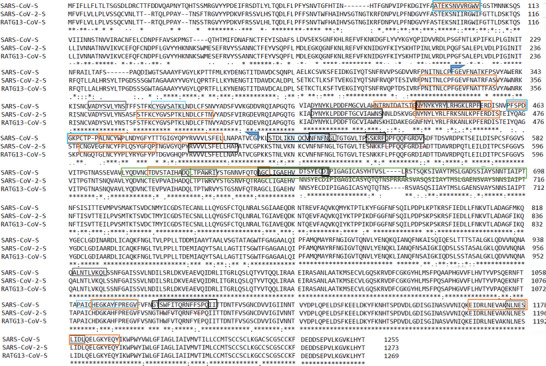
Alignment of SARS‐2, SARS‐1, and RaTG13 structural S‐protein sequences, as generated by ClustalW. The black and blue boxes represent T‐cell epitopes that bind in humans and mice, respectively. The thin and bold boxes represent experimentally immunogenic cytotoxic T‐cell/MHC‐I epitopes and CD4^+^ T‐cell/MHC‐II epitopes, respectively. The orange boxes show experimentally immunogenic or neutralizing S‐RBD B‐cell epitopes, while the green boxes show experimental immunopathological sequences.

Several viral proteins, or their domains/peptidic fragments, inhibit the aforementioned IFN‐1 production and signaling pathways. Therefore, the incorporation of such fragments/epitopes as vaccine antigens should be avoided to prevent training the immune system to develop a pro‐inflammatory response upon viral infection. SARS‐1 NSP‐1,^[^ ^69^, ^70^, ^71^, ^72^ ^]^ NSP‐3,^[^ ^73^, ^74^, ^75^, ^76^ ^]^ NSP‐7, and NSP‐15,^[^ ^74^ ^]^ as well as ORF‐6,^[^ ^74^, ^77^, ^78^ ^]^ M‐protein,^[^ ^79^ ^]^ and N‐protein,^[^ ^74^, ^78^, ^80^, ^81^, ^82^, ^83^ ^]^ were found to inhibit IFN‐I production^[^ ^70^ ^]^ (Figure 4); most of these protein sequences are highly conserved in SARS‐2 (**Figure** **5**). NSP‐1, through degradation of host mRNAs,^[^ ^70^, ^71^ ^]^ and ORFs‐3a and ‐6, through blocking IFNARs,^[^ ^74^, ^77^, ^78^, ^84^ ^]^ inhibit signaling for IFN‐1 amplification in infected host cells. M‐protein inhibits TRAF‐3 complex formation, while N‐protein, NSP‐3, and ORF‐3a and ‐6 inhibit phosphorylation of IRF‐3 as a key step in IFN‐I production.^[^ ^73^, ^74^, ^75^, ^76^, ^77^, ^78^, ^79^, ^80^, ^81^, ^82^, ^83^, ^84^, ^85^, ^86^ ^]^ Moreover, NSP‐1, N‐protein, E‐protein, S‐protein, and ORF‐3a, ‐3b and ‐7a upregulate the expression of pro‐inflammatory chemokines and cytokines from infected host epithelial cells by activating NF‐*κ*B. These proteins are highly similar (80–90%) to their counterparts in SARS‐2, except for ORF‐3b, which is absent in SARS‐2. It is likely that they play similar immunopathological roles in SARS‐2 infections.

### 2.2 ACE2‐RBD Binding‐Induced Immunopathology

ACE2 plays a major role in tissue repair, homeostasis, and fluid balance, while increased levels of Ag‐II are associated with acute tissue damage.^[^ ^31^ ^]^ It was found that administration of SARS‐1 S‐protein downregulates ACE2 expression, induces inflammatory responses, and exacerbates acute lung injuries in vivo in mice.^[^ ^87^ ^]^ Low ACE2 expression levels lead to an increase in Ag‐II, which increases blood pressure, thrombus formation, reactive oxygen species, and upregulation of matrix metalloproteinase‐2 and ‐9, as well as inflammatory cytokines, for example, IL‐6.^[^ ^88^, ^89^ ^]^ Increased levels of Ag‐II in the blood are associated with acute pulmonary tissue damage and inflammatory cytokine (e.g., IL‐6) release. These effect were reversible upon addition of recombinant rACE2, which resulted in lower IL‐6 and Ag‐II levels and an increase in angiotensin 1–7 levels.^[^ ^90^ ^]^

Clinical evidence supports the role of ACE2 in tissue infection tropism and severe illness sequelae in both SARS‐1 and SARS‐2 infections. Loss of RAS system balance, fluid balance, and high blood pressure have been reported in severe COVID‐19 illness.^[^ ^91^, ^92^, ^93^ ^]^ All COVID‐19 mortalities have shown pneumonia and acute respiratory distress syndrome, characterized by severe inflammation and diffuse alveolar damage; a third of fatalities have additionally displayed pulmonary artery emboli.^[^ ^89^, ^94^ ^]^ The damage afflicted in the lower respiratory tract and heart tissue of patients, especially in the ventricular myocytes, can result in hypoxic respiratory failure and ventricular remodeling leading to deteriorating condition and increased risk of death.^[^ ^95^ ^]^ The transcription levels of viral RNA in heart tissue of SARS‐deceased patients were associated with a decrease in tissue ACE2‐expression, that is, below normal levels, an increase in macrophage infiltration, and a higher extent of tissue damage.^[^ ^33^, ^95^ ^]^ Therefore, inhibiting SARS‐S binding to ACE2 should not only relieve the symptoms but also arrest the replication cycle of the virus by interrupting the fusion process, thus protecting deeper tissue and systemic invasion.

### 2.3 SARS‐1 and SARS‐2 Proteins and their Fragments as Candidate Antigens

S‐protein is the most surface‐exposed SARS‐2 protein. It is also the most immunogenic, as 90% of antibodies (Abs) in patients infected with SARS‐2 are directed against it. However, when full‐length S‐protein or DNA‐encoding S‐protein were employed as vaccine antigen against SARS‐1 in low dose, pro‐inflammatory responses were observed in mice and ferrets.^[^ ^96^ ^]^ Furthermore, inflammation at the immunization site and within the liver was reported after injection of Vaccinia Ankara virus bearing S‐protein in ferrets, which was not present in blank virus negative controls.^[^ ^97^ ^]^ The inflammatory component of the S‐protein sequence explains the high immunopathological scores in preclinical trials associated with S‐protein‐based subunit vaccines, and even S‐protein‐encoded RNA as vaccine antigen against SARS‐1 in mice, ferrets, and nonhuman primates.^[^ ^96^, ^98^, ^99^ ^]^ Since a variety of studies have shown that S‐protein induces immunopathological reactions when used as antigen, studying the immunopathology of this protein and its fragments is critical for the development of a safe and effective peptide vaccine.

SARS‐1‐S stimulated the production of IL‐6, TNF‐*α*, and IL‐8 in lung epithelial cells, in peripheral blood monocytes, and in murine macrophages, in vitro through AP‐1 and NF‐*κ*B pathways (Figure 4).^[^ ^100^, ^101^, ^102^ ^]^ Two inflammatory sequences were identified by spiking these cells in vitro, followed by analysis of the cytokine mRNA produced via qPCR. The first sequence, at position 324–488, overlapped with RBD and the second, position 609–688, was located at the *C*‐terminus of the RBD at the S1/S2 site. The sequences were not mapped further to minimal pathological epitopes.^[^ ^100^, ^101^, ^102^ ^]^ Therefore, while immunopathological when whole, it is possible that only part of the sequence is dangerous. Controversially, there is no evidence that peptides derived from the RBD are immunopathological.^[^ ^101^ ^]^ The RBD of SARS‐1‐S and SARS‐2‐S (75% similar) have proven to be protective, immunogenic, and inclusive of neutralizing epitopes.^[^ ^28^, ^103^, ^104^ ^]^ Immunization against SARS‐1 using the RBD as vaccine antigen provided potent Abs in mice that neutralized SARS‐2‐S protein in vitro.^[^ ^14^, ^28^, ^105^ ^]^ In a later study, RBD‐based vaccine did not cause immunopathology in animal models.^[^ ^28^, ^104^ ^]^ Thus, an alternative explanation of the immunopathological role of the S‐protein 324–488 sequence is needed.

Sequence binding may induce downregulation of host ACE2.^[^ ^106^ ^]^ Suppression of the MAPK‐NF‐*κ*B pathway is a normal function of ACE2 in rat lungs, where downregulation increases inflammatory cytokine production (Figures 4 and 5).^[^ ^87^, ^107^ ^]^ This effect might explain the immunopathology of the S‐protein 324–488 sequence, rather than it having a direct effect on NF‐*κ*B or AP‐1, because it overlaps with 83% of the RBD (Figure 5). This suggests that RBD, and even more so, RBD‐peptide fragments, may be safer and more effective antigens compared to full‐length S‐protein.

**Figure 6:**
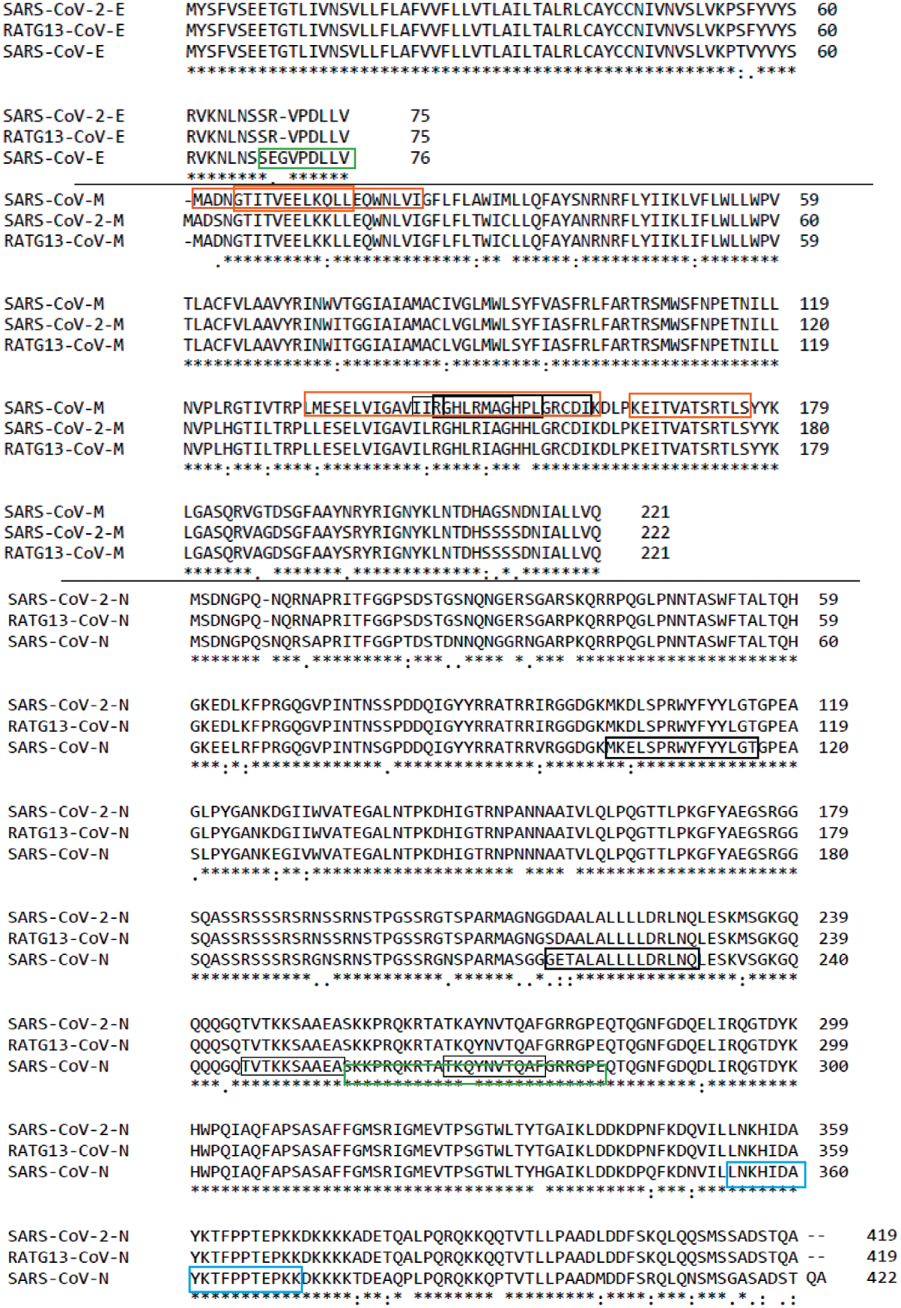
Alignment of SARS‐2, SARS‐1, and RaTG13 structural E‐, M‐, and N‐proteins, as generated by ClustalW. The black and blue boxes represent T‐cell epitopes that bind in humans and mice, respectively. The thin and bold boxes represent experimentally immunogenic cytotoxic T‐cell/MHC‐I epitopes and CD4^+^T‐cell/MHC‐II epitopes, respectively. The orange boxes show experimentally immunogenic B‐cell epitopes, and the green boxes are experimental immunopathological sequences.

Other SARS proteins could also prove unsafe for use as antigen. For example, envelop protein (E‐protein) has a short amino acid sequence of less than 100 residues, but it has several pathogenic functions that contribute to the virulence of SARS‐1. The short octamer (SARS‐1‐E: ^67^SE**G**V**P** D **L** L **V** ^75^) at the *C*‐terminus of E‐protein (**Figure** **6**) is essential for ion channel activity in host cells, thus contributing to virulence^[^ ^108^, ^109^ ^]^ and the induction of pro‐inflammatory cytokines.^[^ ^46^, ^110^ ^]^ The E‐protein octamer is similar to its counterpart in SARS‐2 (CTD of E‐protein: ^68^SRV**P** D **L** L **V** ^76^), and most of the pathology‐related residues are the same between these two proteins (Figure 6). Mutant‐SARS‐1 lacking the E‐protein octamer did not trigger immunopathology in mice after infection.^[^ ^46^, ^74^ ^]^ This suggests that the remaining E‐protein sequence might be safe as a vaccine antigen (Figure 6). However, E‐protein also induced T‐cell apoptosis when co‐incubated with Jurkat T‐cells in vitro.^[^ ^111^ ^]^ T‐cell apoptosis is known to cause a feedback loop in the signaling of inflammatory cytokine production.

Nucleocapsid protein (N‐protein) stimulates inflammatory cytokine production because of its *C*‐terminus amino acid sequence, SARS‐1‐N: ^248^TKKSAAEASKK‐PRQKRTATK**Q**YNVTQAFGRRGPE^281^ (Figure 6). The sequence was not finely mapped further for short immunopathological sequence(s) and it was determined by deletion approach. The N‐protein immunopathological sequence corresponds to a very similar sequence in SARS‐2 located at a similar position, SARS‐2‐N: ^247^TKKSAAEASKKPRQKRTATK**A**YNVTQAFGRRGPE^280^, which differs by just one residue (Q^276^A) in SARS‐2 (Figure 6).^[^ ^80^ ^]^ Deletion of the fragment rendered the protein innocuous in vitro. This also suggests that the rest of the N‐protein sequence is safe to include as vaccine antigen, especially since it includes potent cytotoxic T‐cell epitopes.

To date, SARS‐2‐S was found to trigger inflammatory cytokines (IL‐6, IL‐1ß, TNF‐*α*, CXCL‐1, CXCL‐2, and CCL‐2) production by human and mouse macrophages in vitro via TLR‐2 and TLR‐4 mediated activation of NF‐*κ*B pathway. While SARS‐2‐E was found to induce inflammatory cytokines (CXCL‐10, CCL3, CXCL‐1) production via TLR‐2/MyD88 activation of NF‐*κ*B pathway in mice.^[^ ^112^, ^113^, ^114^ ^]^ However, the immunopathological sequences responsible for this immunopathological responses have not been identified/mapped. In summary, structural proteins (S, E, M, and N proteins) are of particular interest as vaccine antigens. However, they can induce inflammatory responses (S‐protein), inhibit IFN‐1 production (M‐proteins), or both (E‐ and N‐proteins) (Figure 6). The inhibition of IFN‐1 and upregulation of pro‐inflammatory responses continues with release/translation of the protein antigen; this could last longer for DNA‐ and RNA‐based vaccines compared to protein vaccines. Moreover, the effect would be more locally enhanced at the site of injection. Tseng et al. 2012^[^ ^96^ ^]^ reported that intramuscular administration of either inactivated SARS‐1 or S‐protein to BALB/c mice incited lung immunopathology (lung lesions and eosinophilia) post‐infection challenge, despite the generation of neutralizing antibody (nAb) titers and a reduction of viral RNA titers in mouse lungs. Thus, it is worth mentioning that immunopathology is not associated only with local immune responses, in the same way that vaccines are not limited only to the injection site. Therefore, whole SARS proteins should not be used in their native full‐length forms as vaccine antigens. Peptide‐based subunit vaccines can overcome this issue by employing only immunogenic environment‐exposed sequences that produce noninflammatory neutralizing or opsonic immune responses.^[^ ^115^, ^116^, ^117^ ^]^

## 3 The Protective Immune Response

### 3.1 The Importance of Early Adaptive Immune Response Development

The development of an early “adaptive” immune response is essential for avoiding severe illness sequelae, in the alleviation of infection symptoms, and for infection recovery. In contrast, blocking early immune responses associated with macrophage activation proved beneficial in a SARS‐1 lethal mouse infection challenge. The early depletion of macrophages alleviated symptoms and recruited more professional DCs in the lungs, and 100% of mice survived; depletion of macrophages 1 day later resulted in lower survival.^[^ ^64^ ^]^ Alveolar macrophages are involved in the innate immune response associated with the production of inflammatory cytokine storm, which results in fever, coughing, and inflammatory cell recruitment, while professional respiratory DCs help to develop adaptive immune responses faster.^[^ ^118^ ^]^ This suggests that both timing and adaptive immune response type are vital factors for safe and effective recovery from infection.

To initiate adaptive immune responses, APCs need to be activated by pathogen danger signals or vaccine adjuvant, then perform antigen uptake. Antigen uptake by APCs triggers processing of the antigen into short peptides that bind to APCs’ major histocompatibility complex I (MHC‐I) or MHC‐II molecules, allowing presentation on the APC surface. The activation of CD4^+^ T‐cells by MHC‐II‐T‐cell epitope complex is essential for both Th1 and Th2 immune responses. CD4^+^ T‐cells release cytokines and stimulate the maturation of B‐cells into differentiated class‐switched isotypes and phenotypes, including long‐lived plasma cells for immunological memory.^[^ ^116^, ^119^ ^]^ Furthermore, CD4^+^ T‐cells activate antigen‐specific CD8^+^ T‐cells into cytotoxic T‐cells and memory T‐cells that can last for up to 10 years.^[^ ^115^, ^120^ ^]^ Therefore, CD4^+^ T‐cell epitopes are essential for both cellular and humoral responses. On the other hand, antigen recognition by B‐cell receptors is an independent event that is necessary for humoral responses resulting in Ab production.

SARS infection usually takes place through inhalation or mucosal contact with droplets, dry aerosol, or skin contaminated with the virus. The onset of symptoms usually occurs within 5 days of infection.^[^ ^121^ ^]^ SARS‐2 replicates in type‐II pneumocytes in the lungs, and the innate immune system responds with localized inflammation, which results in the typical COVID‐19 symptoms. Further replication and translation of viral proteins suppresses IFN‐I production, while amplifying pro‐inflammatory cytokine production. This generates the cytokine storm that exacerbates the initially mild symptoms (Figure S1, Supporting Information). In severe cases, lymphocytes in the plasma decrease 2–3 days after infection. Consequently, lymphopenia (<500 cell per mL) in SARS‐2 infection cases correlates with illness severity. In the most common cases, T‐cell plasma count is initially low at around 200 cells per mL, but doubles, or even quadruples 10–16 days post disease onset, marking the beginning of the adaptive immune response (Figure S1A,B, Supporting Information). Response titers to S‐protein fragments vary during infection; anti‐S2 subunit titers are higher than anti‐RBD or anti‐S1 Abs^[^ ^122^, ^123^ ^]^ 10–14 days following the onset of symptoms. In recovered patients, nAb levels increased slowly from day 15 (post‐onset), and patients developed high anti‐S‐protein‐specific IgG titers (110 ± 10 U mL^−1^) by day 22 (post‐onset).^[^ ^50^, ^124^ ^]^ During recovery, usually 16–22 days post onset, monocyte counts increase, CD4^+^ and CD8^+^ T‐cells start to decrease in peripheral blood (Figure S1A,B, Supporting Information), and mature Ab‐producing‐B‐cell populations expand in peripheral blood. The detection and the increase of neutralizing Abs coincides with the resolution of symptoms,^[^ ^121^ ^]^ which suggests a highly protective role of humoral immunity. In contrast, deceased COVID‐19 patients show T‐cell exhaustion and a high percentage (>40–60%) of immature B‐cells around 24 days after onset.^[^ ^125^ ^]^ Thus, both cellular and humoral responses appear to be important for recovery.

In SARS infections, serum levels of pro‐inflammatory cytokines, especially IL‐6, increase from normal (17 pg mL^−1^) to high levels (>41 pg mL^−1^), with these levels correlating to infection severity (Figure S1C,D, Supporting Information).^[^ ^51^, ^61^, ^126^, ^127^ ^]^ Lymphocyte plasma count correlates negatively with nAb titers. This suggests the prevalence of a pro‐inflammatory response associated with a hyperreactive Th2 response.^[^ ^128^ ^]^ Together, this means that it is necessary to engineer an interleukin profile produced by APCs that switches to the safer humoral response by strategically employing Th1/Th2 balanced adjuvants.

### 3.2 The Role of Specific CD8^+^ T‐Cell Responses

CD8^+^ T‐cells are potentially protective against SARS‐2 infections, as they have been protective against previous SARS‐CoV infections.^[^ ^64^, ^129^ ^]^ In severe COVID‐19 illness, peripheral circulating CD8^+^ T‐cell counts are low (Figure S1B, Supporting Information);^[^ ^127^ ^]^ in general, the severity of illness is inversely correlated with CD8^+^ T‐cell counts. This suggests a protective role of CD8^+^ T‐cells. It was recently reported that antigen‐specific CD8^+^ T‐cell responses are highly protective: 90% of C57BL/6 mice (*n =* 8–16) given SARS‐1‐S‐specific CD8^+^ T‐cells epitopes, in the absence of SARS‐1‐specific CD4^+^ T‐cells or B‐cells, survived SARS‐1 lethal infection challenge compared to none in the negative control group.^[^ ^129^ ^]^ Similarly, the passive transfer of CD8^+^ T‐cell epitope with prematured dendritic cells used to exclusively drive the maturation of CD8^+^ T‐cells resulted in superior protection in mice (100% survival) against lethal infection by SARS‐1.^[^ ^64^ ^]^ Furthermore, in a recent study on COVID‐19 patients with moderate illness severity, the resolution of clinical symptoms and viral clearance mostly coincided with the development of adaptive immune responses: this was highlighted by mature B‐cells and cytotoxic T‐cell population expansion.^[^ ^50^, ^130^, ^131^ ^]^ However, autopsies of COVID‐19 deceased patients have revealed an accumulation of monocytes and T‐cells in infected lung tissue.^[^ ^127^ ^]^ Therefore, it is likely that in severe illness, cytokine storm‐driven chemotaxis draws unprofessional or nonspecific T‐cells to further contribute to an inflammatory apoptotic response in the lungs.^[^ ^127^, ^132^ ^]^

Moreover, T‐cell apoptosis was also reported following in vitro co‐incubation with SARS‐1‐E protein (has 95% sequence similarity to SARS‐2‐E), which attenuates T‐cell responses.^[^ ^111^ ^]^ This was also supported by counts of exhausted or anergic cytotoxic T‐cells, which correlated positively with disease severity.^[^ ^57^, ^127^, ^133^ ^]^ However, in a recent study, the depletion of CD8^+^ T‐cells did not affect viral clearance or immunopathology, while depletion of CD4^+^ T‐cells delayed viral clearance significantly and resulted in severely exacerbated immunopathology. In contrast, passively immunized mice had reduced viral titers, even with CD8^+^ T‐cell depletion.^[^ ^118^ ^]^ Ultimately, this shows the importance of the humoral response for SARS protection and recovery.

Despite the secondary importance of cytotoxic T‐cell responses in protection compared to humoral responses, memory CD8^+^ T‐cells may still play an important supporting role. Therefore, long‐lived cellular immune responses need to be analyzed. Cellular responses against conserved epitopes, for example, N‐protein, remained for 17 years in SARS recovered patients.^[^ ^134^ ^]^ True memory CD8^+^ T‐cell maturation is driven not only by exposure to antigens but also by APC‐expressed Th1 cytokines (IL‐2, IL‐15, and IL‐18).^[^ ^135^ ^]^ Thus, cellular responses are very long‐lasting via memory T‐cells against viral infections, especially against common conserved epitopes,^[^ ^134^ ^]^ and they can play a synergistically protective role as demonstrated in humanized human leukocyte antigen system (HLA) transgenic mice against SARS‐1.^[^ ^64^, ^129^ ^]^ Therefore, it is vital to 1) include interleukin analysis in future animal studies of vaccines, 2) carefully select adjuvants to exploit cellular immune responses besides humoral immunity, and 3) include T‐cell epitopes in peptide‐based vaccine development to improve the protective capacity of peptide vaccines. This also generates long‐lasting effects more productively, especially in line with recent findings regarding disappearing neutralizing Ab titers within few weeks of recovery of COVID‐19 patients, suggesting the potential for reinfection.^[^ ^136^ ^]^

### 3.3 Humoral Responses

**Figure 7:**
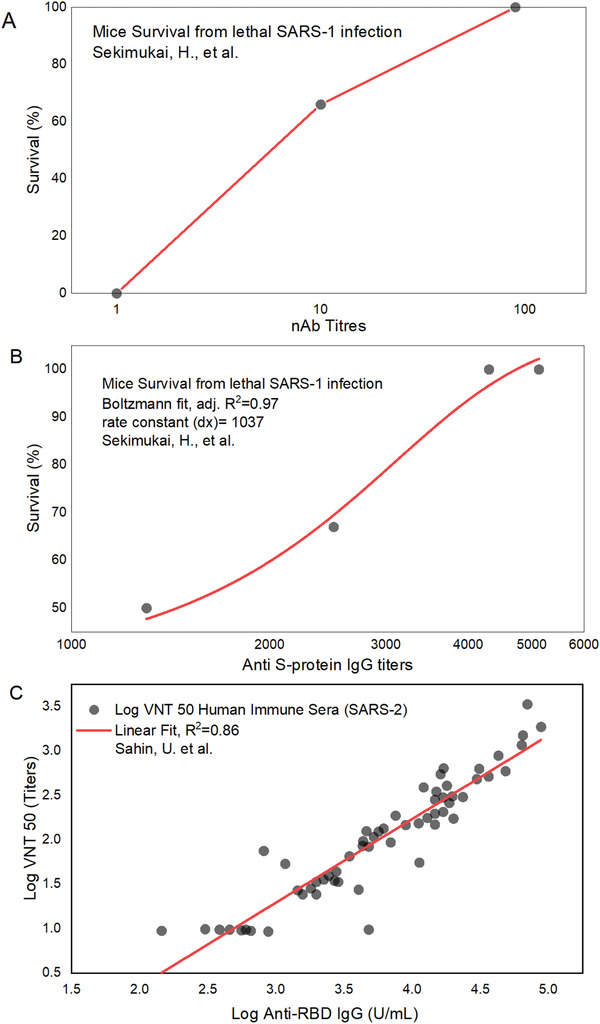
Dose response curves for A) mean IgG neutralizing Ab (nAb) titers and B) mean total anti‐S‐protein IgG titers expressed as the mean reciprocal serum dilution (*n* = 6–7 mice per group). Both are plotted against mouse survival, following the administration of SARS‐1‐S‐protein as antigen with TLR‐3‐ligand (poly I:C adjuvant) or without adjuvant.^[^ ^154^ ^]^ Log_10_ of the total anti‐S‐protein titers of 3.5 offers 100% protection in (B), which was equivalent in protective efficacy to neutralizing Ab (nAb) log_10_ titers of 2 in (A), as both plots are from the same serum samples, (C) relationship between human immune sera against SARS‐2 and neutralization, fitting log IgG concentration and log SARS‐2 neutralization titer 50%, gave  LogVNT_50_ =   − 1.53 + 0.94 · Log IgG_Anti − RBD_, *n* = 59, R^2^ = 0.86.^[^ ^149^, ^150^ ^]^

**Figure 8:**
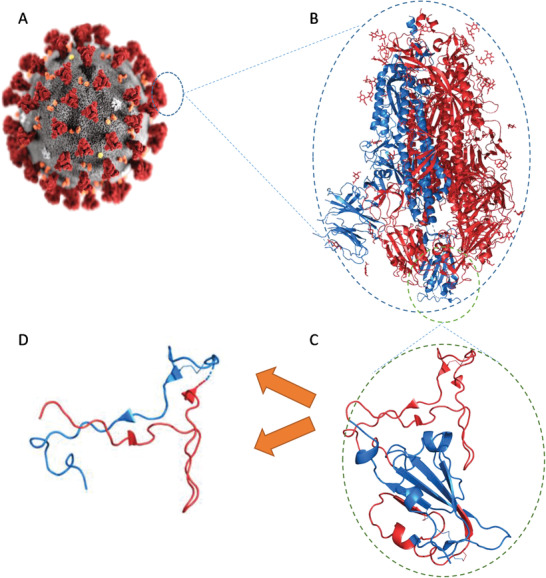
Schematic representation of subunit vaccines’ evolution from A) whole‐pathogen (SARS‐2), to B) subunit protein (S‐protein), to C) immunogenic protein fragment (RBD), and finally D) peptide‐based vaccine antigens (red, pep1; blue, pep2).

Humoral responses are the primary means of protection against SARS‐1 and SARS‐2 infections.^[^ ^50^, ^130^, ^131^ ^]^ Moreover, standardized convalescent patient plasma is used as a safe and effective treatment against SARS‐2 infection.^[^ ^137^, ^138^ ^]^ A correlation (R = −0.69) between neutralizing anti‐RBD Ab titers and viral RNA titer reduction in bronchoalveolar lavage fluid was recently established in rhesus monkeys infected with SARS‐2.^[^ ^139^, ^140^ ^]^ In convalescent patients, anti‐RBD IgG titers were at the level of 10^5^, the titers levels correlated (*R* = 0.64) with 50% neutralization capacity of sera, which were 50% neutralizing in outpatients from as low as about 40‐fold dilutions.^[^ ^141^ ^]^ This substantiates the major contribution and protective potential of humoral immunity though Ab neutralization mechanism. Moreover, plasma of convalescent patients is effective against SARS‐1 infections only when they carry virus nAbs.^[^ ^128^, ^137^, ^141^, ^142^ ^]^ SARS‐1 viral titers reduced dramatically as nAb titers increased, even when passively supplied in mice,^[^ ^143^ ^]^ monkeys, and humans.^[^ ^144^, ^145^ ^]^ Moreover, passive immunization of mice with neutralizing anti‐S‐protein mouse‐adapted Abs (S3.1) derived from SARS convalescent patients, in the range of 0.2 to 1 mg, protected mice from lethal SARS‐1 infection challenge.^[^ ^146^ ^]^ Further, when patients were treated with immune sera, nAbs dose of about 1 mg lowered the SARS‐1 viral load 10^3^ folds^[^ ^142^, ^147^, ^148^ ^]^ without the presence of the cellular immune component. The survival of mice infected with SARS‐1 correlated with anti‐S‐protein IgG titer levels and this relationship showed higher dependency with neutralizing Ab (nAb) titers (**Figure** **7**). Anti‐SARS‐2‐S nAbs were found to be effective, even at levels as low as 7 ng mL^−1^, as determined in convalescent patient plasma.^[^ ^123^ ^]^ Further, anti‐SARS‐2‐RBD IgG (U mL^−1^) in immune patient sera correlated very strongly with virus neutralization titers (VNT) (*R^2^* = 0.86, *n* = 59) in vitro LogVNT_50_ =   − 1.53 + 0.94 · Log IgG_Anti−RBD_ .^[^ ^149^, ^150^ ^]^ This shows that anti‐RBD IgG concentrations up to 1.53 U mL^−1^ were ineffective, while above this value log 50% neutralization titer value increased proportionally to log anti‐RBD IgG concentration (U mL^−1^) (Figure 7C). The Abs were directed against the critical binding domains of S‐RBD (Figure 8).^[^ ^141^ ^]^ In SARS‐2 infection, a high proportion of Ab titers are usually directed against the S2 subunit. These Abs may not be neutralizing; however, they might be protective through the Ab‐dependent cytotoxicity effect (ADCC) pathway.^[^ ^103^, ^128^, ^151^, ^152^, ^153^ ^]^

## 4 Vaccine Development

### 4.1 Whole S‐Protein‐Based and RBD‐Based Vaccines

S‐protein is the main antigenic target for SARS‐2 vaccine development; however, the protein is metastable. Therefore, it is important to stabilize its “active” conformation and prevent premature enzymatic degradation. A prefusion‐stabilized S‐protein developed using molecular clamping was recently employed as vaccine antigen.^[^ ^155^ ^]^ Several copies of the heptad repeat region were employed to clamp the termini of each S‐protein protomer's soluble ectodomain (Figure 1). This ensured that conformational epitopes were correctly recognized by B‐cell receptors, in order to produce potent, effective, opsonizing or neutralizing Abs. To prevent priming by host protease enzymes, proline/glycine substitutions at cleavage sites S1/S2 and S2ʹ are necessary.^[^ ^18^, ^123^ ^]^ Alternatively, instead of whole S‐protein, the RBD region can be employed as antigen. An RBD‐based vaccine (UB‐612) is currently in Phase 1 clinical trials (COVAXX Company). UB‐612 contains RBD‐protein combined with T‐cell epitopes from S2‐subunit, N‐protein, and M‐protein. These T‐cell epitopes can bind to human MHC‐I and MHC‐II to help provide synergistic cellular and neutralizing humoral responses. The clinical trial results of both S‐protein and RBD‐based vaccines are expected to be published in 2021, clinical trial identifier number: NCT03595995.

Whole S‐protein‐based vaccines generate protective responses against SARS‐1 and SARS‐2 infections in mice and monkeys, even without adjuvant (**Table** **1**). However, immunization with the vast majority of these vaccines resulted in exacerbated immunopathology, even when adjuvant‐free S‐protein‐based vaccines were used.^[^ ^96^, ^97^, ^156^ ^]^ Surprisingly, vaccine administered with poly I:C and Th1‐type adjuvant was the exception.^[^ ^154^ ^]^ This can be explained by the less inflammatory nature of Th1 humoral responses. Histopathological examination showed immunopathology represented by eosinophil infiltration and lesions in the lungs of mice. This suggests that immunopathological sequences (Figure 5) compromise the safety of S‐protein‐based vaccines. However, since the immunopathology is cytokine driven, adjuvants may play an important role in modulating interleukin profile‐driven immune responses. Thus, strategic use of adjuvant that switches the immune response from hyperactive Th2 to a safer humoral Th1 response may improve vaccine safety and efficacy.^[^ ^154^, ^157^ ^]^ RBD‐based vaccines have generally been less immunogenic compared to full‐length S‐protein vaccines, but they are capable of producing nAbs and are associated with lower adverse events, that is, immunopathology. Although, several studies showed potent neutralization of Abs generated against RBD‐based vaccine antigens, but immunopathology was not well investigated in preclinical studies against SARS‐1,^[^ ^158^, ^159^, ^160^ ^]^ or against SARS‐2 to date.^[^ ^161^ ^]^

**Table 1:** Whole virus, whole S‐protein, and RNA‐encoding whole S‐protein vaccines against SARS‐1 and SARS‐2

Antigen–adjuvant	Animal model; Route (schedule); and Challenge	Immunopathology	Efficacy	Ref.
‐	(#) Day (D)	—	%	—
SARS‐CoV‐inactivated whole virus vaccine				
Double‐inactivated SARS‐1 (DIV) (+/− alum) [0.25–2 mg]	Female BALB/c mice (*n =* 12–13) IM (D 0, 28) 10^6^ TCID per 60 uL IN, C (D 56)	Th‐2‐type immunopathology erupted after challenge in all groups: **Lesion** scores were lower with alum, in decreasing order: PBS = S‐protein > DIV ≥ PIV. **Eosinophil** % were higher than for whole virus vaccines, in decreasing order: PIV ≥ DIV > S‐protein. The same pathology occurred in female C57BL/6J mice.	Decreasing order of nAb titers: DIV ≥ S‐protein (titers = 2^9^) > PIV. NAb titer response increased with vaccine dose, and was generally higher with alum for all vaccines. Viral titers with alum in increasing order: DIV (10^1.5^) < PIV (10^1.7^) < S‐protein (10^2^); Viral load titers after challenge dropped further with alum for all vaccines at similar antigen doses (PBS control titers = 10^6.5–7.5^). Higher vaccine doses did not improve protection or increase immunopathology. No differences in viral titer reduction were observed with DIV and PIV. Female C57BL/6J mice were also tested in parallel; similar nAb titer responses were generated with these vaccines.	^[^ ^96^ ^]^
Propionolactone‐inactivated SARS‐1 (PIV) (+/− alum)				
S‐protein +/− alum [3 or 9 mg]				
SARS‐CoV‐2‐inactivated whole virus vaccine				
BBV152 inactivated SARS‐2 Dose of 3 µg, or 6 µg with alum and/or TLR‐agonist 7/8 as adjuvants (imidazoquinoline) in 0.5 mL	Human phase 1/2 clinical trial (*n* = 375) IM (D 0, 14) Placebo control, only alum (*n* = 75)	A controlled dose‐escalation double‐blind study was conducted using adjuvanted inactivated whole SARS‐2 virion as vaccine. Mild to moderate systemic and local adverse events were reported in 17% and 21% in 3 µg dose group and 6 µg dose group with both adjuvants, while 14% and 10% of participants reported local and systemic adverse events in 6 µg dose group with alum and alum only group, respectively, including headache, nausea vomiting, fever, and fatigue. However, one serious adverse event of viral pneumonitis was present among 6 µg dose group with alum. The vaccines with various similarly resulted in generation of anti‐RBD IgG titers 10^3.5^, and anti‐N‐protein IgG titers of 10^3.5^. Live virus neutralization assays showed similar nAb titers of 300 against three different SARS‐2 strains (original strain and D614G mutation), for all vaccine candidates compared to negative control. Live virus microneutralization assay and plaque reduction assays evaluated the 50% neutralization at serum dilution of 90‐85 folds for all vaccines. Recently, hamsters (*n* = 6) were IM‐immunized thrice with, alum/imidazoquinoline–adjuvanted, (3 µg) dose of BBV152 and IN infected with SARS‐2 (10^4.5^ TCID_50_). The vaccine generated nAb titers of 10^4.5^ and resulted in 10^6^‐fold reduction of viral RNA titers in nasal washes by day 7 post‐infection without showing histopathological symptoms ^[^ ^170^ ^]^		^[^ ^171^ ^]^
SARS‐1 subunit S‐protein				
Live‐attenuated measles‐bearing SARS‐1‐S‐protein (MV‐S) [10^5^ TCID_50_]	CD46‐IFNAR^−^ mice (*n =* 6) IP (D 0, 21) SARS‐1 10^5^ PFU IN, C (D 77)	Live‐attenuated MVs bearing S‐protein induced a Th1‐biased response with higher IgG2a (10^4‐5^) to IgG1 (10^3.5^) ratio of 5 and 10 for MV‐S and MV‐S_ecto,_ respectively, while alum‐adjuvanted S_ecto_ induced a Th2‐biased response with IgG1 (titers = 10^4.5^) and low IgG2a (titers = 10^2.5^), thus a ratio of about 0.01. nAb titers against MV‐S were 10^3^ and 10^2.5^ for both MV‐S_ecto_ and alum‐S_ecto_, and nAbs reduced viral titers ≤ 10^2^ in all groups compared to PBS control viral titers of 10^4.5^. Therefore, both Th1 and Th2 responses were adequately protective.		^[^ ^156^ ^]^
MV bearing soluble S‐protein ectodomain (MV‐S_ect_) [10^5^ TCID_50_]				
Soluble S‐protein ectodomain [2 µg] with alum [50 µg]				
Modified Vaccinia Ankara‐bearing SARS‐1‐S‐protein (10^9^ pfu per ferret)	Ferrets (*n =* 3) IP and SC (D 0, 14) SARS‐1 10^6^ PFU C (D 28)	The vaccine produced anti‐S‐protein nAb titers of 0.2–0.4 × 10^2^ pre‐challenge. Three weeks post‐challenge, the titers rose to 0.6–1.3 × 10^3^_._ After challenge, liver enzyme blood levels were highly elevated compared to infected blank Vaccinia virus or the PBS groups. This indicates an inflammatory response due to S‐protein, and S‐protein potentially interacted with hepatocyte ACE2. One‐month post‐challenge, viral RNA in blood and pharyngeal swabs was not detectable. The vaccine induced inflammation in liver tissue that was mild compared to the challenge control group.		^[^ ^97^ ^]^
SARS‐2 subunit S‐protein				
S1‐subunit [35 µg] S‐protein (wild‐type) [35 µg] S‐protein (proline substituted) [35 µg] RBD [100 µg]	BALB/c mice (*n =* 5) IM (D 0, 7, 28) alum or CFA Cynomolgus macaques (*n =* 2) IM (D 0, 14, 42) [20 µg]	The authors expressed S1‐subunit, S‐protein (wild‐type or proline‐substituted) and RBD in insect cells. S1‐ and S‐proteins assumed trimeric form in solution, while RBD was monomeric. RBD and both forms of S‐protein induced high titers (log_10_ > 3), while S1‐subunit had low immunogenicity. Although, recent reports observed the opposite, showing that S1 is superior to RBD.^[^ ^172^ ^]^ This may be explained by altered conformation after insect cell expression. The 10 and 100 µg doses of RBD were comparable in mice with CFA. Proline substituted S‐protein was most immunogenic, even at a 1 µg dose in mice. Neutralization of macaque sera immunized with proline‐substituted S‐protein was effective even at 10^4^‐fold dilution, and 40‐times stronger compared to convalescent patient sera.		^[^ ^161^ ^]^
SARS‐1 DNA‐ and RNA‐based encoding protein antigens				
DNA‐encoding M‐protein (pcDNA 3.1 vector)	SCID‐hu mice injected with healthy human lymphocytes IM (D 0, 7, 14)	M‐ and N‐proteins induced a CD8^+^ T‐cell response in hu‐mice, while neutralizing Abs were produced only against M‐protein. The authors proposed combining M‐ and S‐proteins in vaccines for a potentially synergistic effect. Convalescent patient sera could bind to expressed proteins.		^[^ ^173^ ^]^
DNA‐encoding N‐protein (pcDNA 3.1 vector)				
SARS‐2 RNA‐based encoding protein antigens				
Adenovirus serotype 26 (nonreplicating) vector with RNA‐encoding SARS‐2‐S (wild‐type or proline‐substituted) (Ad26‐S)	Rhesus macaques (*n =* 4–5) IM, single dose IN, C (42) SARS‐2 10^5^ TCID	A preclinical study of immunogenicity and safety of Johnson and Johnson's vaccine. Ab responses protected against viral infection challenge. The best results were achieved by RNA encoding the proline‐substituted S‐protein; this reduced viral titers in nasal swabs and in bronchoalveolar lavage fluid to 10^1.7^, which is the detection limit, by the day 4 post‐challenge, compared to 10^4.9^ in the negative control group. Neutralizing Ab titers correlated with protection and a reduction of viral RNA titers. The responses were Th1‐biased and Elispot showed mature IFN‐*γ* ^+^, CD8^+^, and CD4^+^ T‐cells. Recently, hamsters (*n* = 10) were IN‐immunized twice with (10^7^ copies /mL) dose of a related Ad26‐S vaccine and IN infected with SARS‐2 (2.5 × 10^5^ PFU). The vaccine generated nAb titers of 10^3.2^ and resulted in 10^4^‐fold reduction of viral RNA titers in nasal washes and in lung tissue, by day 8 post‐infection.^[^ ^174^ ^]^		^[^^139^, ^140^^]^
Adenovirus serotype 26 and 5 (nonreplicating) vector with RNA‐encoding SARS‐2‐S [10^11^ PFU]	Humans Phase 3 (*n* = 21 977) IM, (D 0, 21) Placebo controlled, buffer (*n* = 5476).	A placebo‐controlled double blind study of immunogenicity and safety of Gam‐COVID‐Vac (Sputnik V) vaccine. After second boost 0.1% of vaccinated participants and 1.3% of placebo group participants were infected with SARS‐2. The overall efficacy was 91.69%. Grade 1 adverse events were common (94% of vaccinated participants), while serious adverse events occurred in only 0.3–0.4% of vaccinated or control groups. The vaccine resulted in mean RBD‐IgG titers of 10^3^, and of mean nAb titers of only 50 measured by live virus microneutralization assay. The vaccine stimulated IFN‐*γ* from PBMCs.		^[^ ^175^ ^]^
mRNA‐1273 Lipid nanoparticle (SM‐102, SM‐3, DSPC, PEG2000‐DMG, cholesterol) dispersion with mRNA‐encoding SARS‐2‐S [25 or 100 µg]	Humans phase 1 (*n =* 10) (NCT04283461) IM (D 0, 28) 25 µg or 100 µg IM (D 0) 250 µg	At the 100‐µg dose, moderate to severe side effects, including pain and erythema at the injection site were observed; moderate side effects were observed at the 25‐µg dose. Ab titers were seroconverted 2 weeks after the first dose. By day 36 to 57 (8–29 days after the second immunization on day 28), nAb titers of 10^2^ and total anti‐S‐protein titers of 3 × 10^5^ were achieved from the 25‐µg dose, and 2.5 × 10^2^ and total anti‐S‐protein titers of 10^6^ for the 100‐µg dose. In vitro plaque reduction tests and nAb titers for the 100‐µg dose showed neutralization comparable to recovered patients’ sera. The responses were Th1‐biased and TNF‐*α*, IFN‐*γ*, and IL‐2 were elevated in the following rank order TNF‐*α* > IL‐2 > IFN‐ *γ* showing T‐cell activation, while Th2 interleukins (IL‐4 and IL‐13) were very low.		^[^ ^166^ ^]^
mRNA‐1273 Lipid nanoparticle encapsulating mRNA‐encoding SARS‐2‐S [100 µg]	Humans phase 3 (*n* = 30 420) IM (D 0, 28) Placebo controlled (*n* = 15 210)	Placebo controlled 1:1 trial was conducted. After 120 days post‐vaccination, 185 participant from placebo group and 11 from mRNA vaccinated group were infected with SARS‐2. Vaccine efficacy had overall estimate of 94.1%. Participants (≈90%) had grade 1 local adverse events by the second immunization including 25% and 5% with grade 2 and grade 3 adverse events (pain erythema and swelling), respectively. Further, systemic side effects were exhibited by participants, about 15% and 40% developed grade 3 and grade 2 adverse events (headache fatigue, myalgia, arthralgia, nausea, and chills).		^[^ ^176^ ^]^
ChAdOx1 nonreplicating vector, with DNA‐encoding SARS‐2‐S [2.5 × 10^10^ PFU]	BALB/c (*n =* 5) and CD1 mice (*n =* 8) (IM) Rhesus macaques (*n =* 6) (IM) (D 0) 2.6 × 10^6^ TCID SARS‐2 C (D 28)	The vaccine prevented pneumonia in vaccinated animals and reduced immunopathology. Pneumonia signs lasted longer in control animals. The combined clinical score was half that of the control animals in total. Histological examination showed no pathological signs. However, IL‐6 was increased in vaccinated animals. A single IN vaccine dose elicited humoral and cellular responses. The response was Th1‐dominated, shown by cytokine profile and IgG subclasses, and reduced viral titers in the lungs of both mice and macaques. In mice, splenic T‐cells produced IFN‐g in response to peptide stimulation in vitro. Increased levels of IFN‐g, TNF‐a, IL‐4, and IL‐10 were detected in mice. Anti‐S1 or anti‐S2 subunit Ab titers were in the 10^2–3^ level, while nAb titers were 10^2^, with population expansion of IFN‐g^+^, CD4^+^, and CD8^+^ T‐cells. In pre‐challenge macaques, single immunization with vaccine elicited Ab titers of about 10^3^, with only 5–40 nAb titers. At 7‐days post‐challenge, the viral load was reduced to 10^1.5^, compared to 10^5^ for the control group. IFN‐g, TNF‐a, IL‐6, and IL‐10 were all increased after challenge, but IL‐6 and IL‐10 levels dropped quickly 3 days post‐infection. Recently, ferrets (*n* = 8) were IM‐immunized twice with (2.5 × 10^10^ PFU) dose of vaccine and IN infected with (3 × 10^4^ TCID_50_) SARS‐2. The vaccine generated nAb titers of 320, and resulted in 15‐fold reduction of viral RNA titers in nasal washes.^[^ ^177^ ^]^		^[^ ^168^ ^]^
ChAdOx1 nCoV‐19 nonreplicating vector, with DNA‐encoding SARS‐2‐S [5 × 10^10^ PFU]	Human Phase 1/2 (n≈540) (NCT04324606) IM (0, 28) 18–55 years old	Systemic moderate to severe side effects, including pain, fever, chills, and headache, were prominent in vaccine group compared to placebo group. Side effect were reduced by administering paracetamol. Majority of patients (91%) produced detectable nAb titers after single dose, and all of them after boost. Anti‐spike mean titers were comparable to mild illness convalescent patient sera titers. Live SARS‐2‐entry test showed that 50‐ and 512‐fold diluted patient sera, following second immunization, inhibited virus entry to 100% and 50%, respectively. This efficacy was practically identical for convalescent patients' sera. The vaccine induced 700 SFCs of IFN‐*γ* ^+^ T‐cells per million PBMCs in Elispot after boost compared to 50 in negative control.		^[^ ^157^ ^]^
ChAdOx1 nCoV‐19 (AZD1222) nonreplicating vector, with DNA‐encoding SARS‐2‐S [5 × 10¹⁰ PFU]	Human phase 2/3 8534 volunteers Efficacy against B.1.1.7 lineage Control groups, meningococcal conjugate (*n* = 4290)	The SARS‐2 lineage B.1.1.7 is prevalent in the UK, volunteers were enrolled to evaluate efficacy of vaccine against the outspread SARS‐2 lineage, by evaluation of viral RNA in airway swabs on weekly basis against UK consortium SARS‐2 database. About 1466 out of 8534 volunteers were infected with SARS‐2 (6%). Among sequenced samples of infected participants nAb titers were lower against B.1.1.7 than other lineages, and estimate of efficacies were 70.4% and 81.5% against B1.1.7 and non‐B.1.1.7 lineages, respectively. Live virus neutralization test showed that nAb titers were about 2^6^ versus 2^9^ for B.1.1.7 versus non‐B.1.1.7 lineages, respectively.		^[^ ^178^ ^]^
BNT162b1 RNA‐encoding S‐RBD protein [10, 30, or 100 µg]	Human phase 1/2 (*n =* 12) 18–55 years old IM (0, 20) [10 or 30 µg] IM, single dose [100 µg]	The vaccine induced moderate pain at the injection site in 100% patients inoculated with 30 and 100 µg doses, in addition to fever, chills muscle pain and headache. The side effects were moderate to severe in 100 µg doses, and absent, except for injection site pain, in placebo group. The 10 & 30 µg doses were administered twice. The generated nAb titers mean was 1.9‐ to 4.6‐folds higher than convalescent patient sera. Log anti‐RBD IgG titer were directly proportional to log VNT_50_ neutralization titers with a slope approaching 1, Linear fitting gave LogVNT_50_ = − 1.53 + 0.94 · Log IgG_Anti − RBD_, R^2^ = 0.86, where VNT_50_ is viral neutralization 50% titers. This suggests that a starting log anti‐RBD titer ≥1.53 is required for neutralization to become effective. In phase 3 trials vaccine efficacy was >90%, in 43 538 volunteers, so far only 94 were infected post‐vaccination.		^[^^149^, ^150^^]^
SARS‐1 S‐RBD subunit antigen				
RBD (native) glycosyl [20ug] + alum	BALB/c (4‐6 weeks) SC 2% Alhydrogel	Anti‐RBD (native) nAbs recognized expressed deglycosylated RBDs, especially at higher concentrations to different extents. All RBDs induced titers in the 10^5–6^ level. However, nAb titers varied among the RBDs; deglycosylated RBD‐3 (1.5 × 10^3^) had equivalent neutralizing titers to longer‐length RBD 219‐mer (native), and slightly lower than 193‐mer native glycosylated RBD (2 × 10^3^). This shows that glycosylation is not necessary for a strong response and might slightly shield cryptic epitopes. Immunopathology was not investigated and in vivo challenge was not conducted.		^[^ ^160^ ^]^
RBD‐1 (N1 deglycosyl) [20 ug] + alum				
RBD‐3 (N1 deglycosyl, N13S and N40A) [20 ug] + alum				
RBD 219‐mer [10 ug per mouse] + CFA/IFA	Female BALB/c (*n =* 5) SC (D 0, 21, 42) SARS‐1 5 × 10^5^ TCID C (D 52)	Mice were challenged and serum nAbs were tested for efficacy in vitro using VERO E6 cell pseudovirion entry inhibition. Ab titer production increased from 10^3^ to 10^3.7^ throughout immunizations until pre‐challenge. Both RBDs had a neutralization titer 50% of 10^3^. Two RBD‐derived peptides were found to stimulate CD8^+^ T‐cells (S_T17_^365^KCYGVSATKL^374^, single residue different in SARS‐2: A370P) and helper T‐cells (S_T(h)18_^435^NYNYKYRYLR^444^, three residues different in SARS‐2‐S) in vitro by pulsing peptides; this resulted in the production of IFN‐g, IL‐2, and IL‐4 from T‐cells. The RBD 219‐mer challenge groups at 10‐days post‐infection had nondetectable (<10^2^) viral titers compared to 10^5–6^ in the control (PBS) group. Five standard nAbs against conformational epitopes and a nAb against a linear epitope recognized the shorter RBD 193‐mer on ELISA plates.		^[^^158^, ^159^^]^
RBD 193‐mer [10 ug per mouse] + CFA/IFA				
RBD [20 ug] + MPL	BALB/c (*n =* 5) SC (D 0, 21, 42)	The neutralizing epitopes within RBDs were investigated. Six (I‐VI) conformational epitopes and two linear epitopes were isolated. These epitopes bound to nAbs produced from mice vaccinated with RBD. Conformational epitopes IV and V were highly potent in neutralization activity. The linear epitope S_B19_^442^NYNYKYRYLRHGKLRPFERDISNVPFSPDGK^465^ was moderately neutralizing; it is identical to its equivalent in SARS‐2.		^[^ ^179^ ^]^

Ab, antibodies; C, challenge; CFA, complete Freund's adjuvant; D, day; DSPC, 1,2 distearoyl‐sn‐glycero‐3‐phosphatidyl choline; HLA, human leukocyte antigen system; IFA, incomplete Freund's adjuvant; IM, intermuscular; IN, internasal; IP, intraperitoneal; MHC, major histocompatibility complex; MPL, monophosphoryl lipid A; nAb, neutralizing antibody; PBMC, peripheral blood mononuclear cells; PEG‐2000‐DMG, 1,2‐dimyristoyl‐rac‐glycero‐3‐methoxypolyethylene glycol‐2000; PFU, plaque forming unit; SC, sub‐cutaneous; TCID, tissue culture infective dose 50%.

Whole S‐protein‐based vaccine antigens include deleterious epitopes (Table 1, Figure 5). In SARS‐1 infections, some anti‐S‐protein Abs produced enhanced virus entry into host cells, supporting virulence and the spread of the infection.^[^ ^162^, ^163^ ^]^ Anti‐S‐protein Abs caused an antibody‐dependent enhancement (ADE) effect in macaques upon challenge with SARS‐1 virus. Histopathological examination of the lung tissue showed that Ab‐enhanced infectivity resulted in exacerbated interstitial pneumonia, acute tissue damage, and diffuse alveolar damage.^[^ ^163^ ^]^ The possibility of ADE has also been suggested for SARS‐2.^[^ ^162^, ^164^, ^165^ ^]^ Thus, ADE‐triggering epitopes should be identified and excluded from SARS‐2 vaccines or, as a minimum precaution, vaccines should be tested for potential ADE‐related side effects.^[^ ^165^ ^]^ While using whole S‐protein, or its encoding RNA/DNA as vaccine antigens, is protective, however this is the overall outcome, the vaccines might be more effective with the exclusion of ADE‐triggering sequences, which are not easily detected.^[^ ^164^ ^]^

DNA‐ and RNA‐based vaccines encoding SARS‐2‐S‐protein were employed in nonlive cationic lipid vectors, such as mRNA‐1723, or in live nonreplicating viral vectors, such as adenoviruses AD serotype‐26 or the chimpanzee adenovirus oxford strain‐1 (ChAdOx‐1). DNA and RNA‐vaccines often develop moderate to weak responses because the transfection efficacy of safe, nonlive cationic vectors is low. Moderna Co. developed an RNA liposomal formulation with cationic transfecting lipids (mRNA‐1273) to immunize human volunteers in a Phase 1 clinical trial. The mRNA‐1273 vaccine produced sera with neutralizing Ab titers comparable to convalescent patient sera.^[^ ^166^ ^]^ However, immunization with mRNA‐1273 vaccine also resulted in moderate to severe side effects, including fever, pain, and erythema at the injection site and acute allergic/anaphylactic reactions.^[^ ^167^ ^]^ Live, nonreplicating vectors are generally more effective in achieving high transfection levels, but they are also associated with more severe adverse events.

Chadox‐1 carrying DNA‐encoding SARS‐2‐S has passed clinical trials and approved for human use.^[^ ^157^ ^]^ In reported results of clinical trials, the immunized sera were neutralizing and passed the in vitro neutralization tests, including plaque reduction neutralization assay and the pseudovirion cell entry inhibition test.^[^ ^157^ ^]^ Similar to mRNA‐1273, Chadox‐1 vaccine was highly effective, generated Th1‐biased responses, and potently neutralizing sera. Furthermore, the interleukin profiles of animal tested, including mice and rhesus macaque monkeys, showed reduced signs of immunopathology, including elevated IL‐6, but no pneumonia or diffuse alveolar damage.^[^ ^168^ ^]^ However, immunization with chadox‐1 vaccine has also resulted in side effects, including pain, chills, fever, and malaise.

More recently, Pfizer released another mRNA‐based vaccine product (BNT162b1) that has passed clinical trials and approved for human use.^[^ ^149^, ^150^ ^]^ The mRNAs included encoded RBD in trimeric form loaded into a lipid transfection lipoplex system. According to data from clinical trials, it offered 90% protection after two doses of 30 µg of RNA. The sera of immunized patients had neutralizing Ab titers 1.9–4.6‐fold higher than convalescent patients. The vaccine elicited a Th1‐biased immune response with elevated IFN‐*γ*, as well as humoral and CD8^+^ T‐cell responses.^[^ ^169^ ^]^ However, pain was experienced at the injection site in all patients after the first dose of either 30 or 100 µg, which turned into moderate pain after the second dose. This adverse reaction is partially due to the transfection lipoplex, but also to an inflammatory reaction to the antigen since the placebo generated less adverse reactions (22% of patients). The vaccine also induced muscle pain, chills, and fever after the first dose in a dose‐dependent manner; however, these events were not severe of grade 4.^[^ ^149^ ^]^ In addition, Moderna and Pfizer/Biontech mRNA vaccines induced acute systemic reactions; when group of 64 900 healthy volunteers were immunized, 2% of them developed acute allergic reactions, and 0.02% anaphylactic reactions.^[167]^ The side effects were absent in the placebo group, indicating immunopathology of the encoded RBD antigen. Therefore, while the induction of Th1‐type immune responses is important toward safe vaccine development, it is not sufficient on its own to produce safe vaccine, as demonstrated by these RNA‐based examples.

### 4.2 Epitope Screening Considerations for Peptide‐Based Vaccines

B‐cell epitopes are mostly (90%) conformational, which implies that 90% of epitopes are discontinuous.^[^ ^180^ ^]^ Thus, it is rather difficult to identify continuous peptide epitopes that are both protective/neutralizing and that maintain native structure conformation. The RBD is an obvious target for epitope screening for SARS‐2 vaccine development.^[^ ^103^ ^]^ Epitopes should be a) protein‐surface exposed, b) contain a sufficiently long sequence to maintain conformational features, for example, full helices, to preserve native conformation, and c) contain some but not all of the CBRs necessary to bind RBD to ACE2. This ensures recognition by B‐cell receptors in native conformation. Epitopes should also be tested for their affinity toward ACE2 binding, which should be minimal. Other groups of promising epitopes are those at or near cleavage sites, thus preventing priming by TMPRSS2 or furin action at the S1/S2 or S2ʹ sites.

Focusing immune response against known neutralizing or opsonizing epitopes has been found to be extremely effective in generating protective responses against several pathogens, especially viruses.^[^ ^151^, ^152^, ^181^, ^182^, ^183^, ^184^, ^185^ ^]^ For example, a vaccine antigen triggered Ab response against influenza virus and generated a response that protected more mice from viral infection challenge, compared to the native fusion protein.^[^ ^153^ ^]^ Improved immunogenicity was also observed when a similar approach was employed for gp120 of the HIV virus.^[^ ^151^ ^]^ Therefore, focusing the immune response against fewer, but highly effective or neutralizing epitopes as a general strategy can be an effective approach that affords superior protection. Peptide‐based epitope combined with strong adjuvant that recruits APCs effectively, therefore, is a more direct and often potent approach compared to a large protein‐based antigen that contains hundreds of obsolete epitopes—most of which provide no or undesired immunity.

Exposed epitopes are often more immunodominant compared to less accessible, buried sequences. Insertion of multiple copies of a cryptic epitope at different positions within a fusion protein sequence did not help to improve the immunogenicity of HIV protein‐based vaccine due to a lack of exposure of these epitopes.^[^ ^184^ ^]^ Interestingly, immunodominant epitopes might not always be the most effective antigens for peptide‐based vaccine development. Cryptic epitopes are sequences that are initially less exposed and located at low profile sites that become exposed when structural conformation alterations take place during protein movements (breathing), binding, or functioning. These epitopes often remain highly conserved across pathogen strains and mutations. However, it is highly unlikely that completely buried epitopes could be made more immunogenic by insertion of multiple epitope copies in protein antigen. In contrast, the peptide‐based vaccine approach guarantees exposure of epitopes, and therefore their recognition by B‐cell receptors. Cryptic epitopes, once administered with a strong adjuvant, may neutralize a virus or trigger ADCC mechanisms.^[^ ^141^, ^186^, ^187^ ^]^

**Figure 9:**
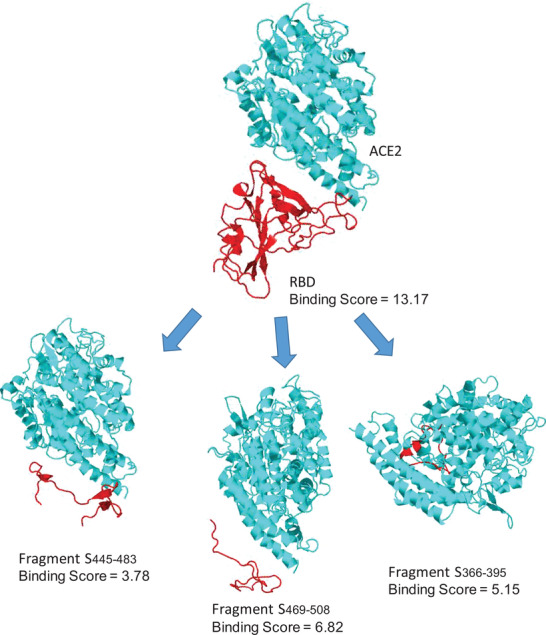
Binding scores using the i‐tasser SPRING server for RBD and several RBD‐fragments to ACE2.^[^ ^188^ ^]^

B‐cell receptors do not recognize whole RBD sequences; instead, they bind much shorter fragments (peptide epitopes). Thus, these epitopes are too small to bind effectively to ACE2, as they do not possess enough CBRs or are conjugated to bulky carriers (often used for peptide epitope delivery), which creates steric hindrance. Therefore, the administered antigen is available for B‐cell receptors without dose reduction associated with binding to ACE2, and without triggering the adverse effects associated with such binding. The binding affinity of SARS‐2‐RBD to ACE2 is around 15 nm.^[^ ^23^, ^42^ ^]^ We explored the binding of peptide fragments of RBD to ACE2 (unpublished data) with the i‐tasser SPRING template server of Michigan University using protein–protein complex interaction reported by Guerler et al.^[^ ^188^ ^]^ When we compared RBD/ACE2 binding to the binding of potentially neutralizing epitopes from the RBM region (pep1: ^445^VGGNYNYLYRLFRKSNLKPFERDISTEIYQAGSTPCNGV^483^), (pep2: ^469^STEIYQAGSTPCNGVEGFNCYFPLQSYGFQPTNGVGYQPY^508^), and one highly conserved cryptic epitope outside of the RBM region but within the RBD sequence (pep3: ^366^SVLYNSASFSTFKCYGVSPTKLNDLCFTNV^395^), the binding scores were 2‐ to 4‐fold lower than full‐length SARS‐2‐RBD; resulting in scores of 3.8, 6.8, 4.7, and 13.2 for pep1, pep2, pep3, and RBD, respectively (**Figure** **9**). Thus, as expected, the loss of several CBRs greatly reduces binding affinity to receptors. Thus, employing RBD or soluble S‐protein as vaccine antigen will result in binding to human cells expressing ACE2, for example, muscles, adipose tissue, and kidney cells,^[^ ^24^ ^]^ which would lead to antigen loss following subcutaneous or intramuscular administration. As such, peptide‐based vaccines offer a very promising approach, which could negate off‐target antigen loss, immunopathological effects, and lung injury due to ACE2 downregulation by S‐protein/RBD binding to ACE2.^[^ ^87^, ^101^ ^]^

Several recent observations support the theory that SARS‐2 behaves similarly to SARS‐1 and that humoral response remains the key to recovery. Similar to SARS‐1 infections, 90% of Abs produced against SARS‐2 in COVID‐19 patients are directed against S‐protein.^[^ ^189^ ^]^ New epitopes specific for SARS‐2‐RBM (has 59% sequence similarity to SARS‐1‐RBM) within SARS‐2‐S‐RBD were mapped and found to overlap within the above mentioned Pep1‐3 sequences.^[^ ^103^ ^]^ However, highly neutralizing anti‐SARS‐1‐S nAbs, such as m396, CR3022, 80R, and S230 were found to be ineffective against SARS‐2, and CR3014 was found to bind to SARS‐2‐S with rather low competitive affinity to ACE2.^[^ ^41^, ^190^ ^]^ Yet, in a recent study, two old anti‐SARS‐1‐S neutralizing Abs were found to bind to SARS‐2‐RBM CBRs noncompetitively with highly neutralizing capacity; they lowered viral load titers by 10^3^‐fold in mice.^[^ ^142^ ^]^ Sera Abs from both SARS and COVID‐19 recovered mice and humans were found to bind to the RBD of S‐protein and block SARS‐2 cell entry.^[^ ^41^, ^147^, ^148^, ^191^ ^]^

The most potent nAbs are mainly directed toward conformational epitopes located in the RBD of SARS‐1‐S,^[^ ^158^, ^159^, ^179^, ^192^ ^]^ which carry CBRs to ACE2 receptors.^[^ ^15^, ^193^ ^]^ SARS‐2‐S binds to ACE2 in an identical manner to SARS‐1‐S, and has similar CBRs. The relative importance of each of the binding residues in SARS‐1‐RBD were determined by single residue mutations (Figures 1C and 5). These residues can be classified into moderately, highly critical, and essential residues for the binding of RBD to ACE2. Single residue alanine substation at T^431^, a moderately critical residue, reduced ACE2 binding to 50%, while single residue mutation at any of the essential residues K^390^, D^429^, or N^473^, reduced binding by almost 100%. Moreover, single mutation in any of the highly critical residues: R^426^, F^485^, Q^492^, Y^494^, D^454^, or R^495^, reduced binding by 90%. Recently, critical residues for SARS‐2‐S were aligned to the SARS‐1‐S 3D structure (Figures 1 and 3).^[^ ^193^ ^]^ Glycosylation was found to be nonessential for ACE2 binding^[^ ^194^ ^]^ and, therefore, glycosylation of selected epitopes is not required unless it is employed to maintain structural conformation.^[^ ^194^ ^]^ Targeting several CBRs in RBD sequences should prevent mutant strain escape from neutralization. Such critical residues can be included in one‐ or two‐long peptide epitopes (G^446^, Y^449^, Y^453^, L^455^, F^456^, and A^475^) as in the Pep1 sequence, or (A^475^, F^486^, N^487^, Y^489^, Q^493^, G^496^, Q^498^, T^500^, N^501^, G^502^, and Y^505^) as in the Pep2 sequence (Figure 9).

Anti‐RBD Abs constitute about 90% of all the neutralizing Abs against SARS infection; thus, they are considered to be highly protective and neutralizing.^[^ ^195^ ^]^ Other neutralizing epitopes are located in the N‐terminal domain of the S‐protein.^[^ ^196^ ^]^ Unfortunately, it was recently observed that anti‐RBD neutralizing Ab titers decay rapidly within a few weeks (*t*_1/2_ of 49 days) after recovery from SARS‐2 infection.^[^ ^195^, ^197^, ^198^ ^]^ Several cases of reinfection have been reported.^[^ ^199^, ^200^ ^]^ Naturally acquired immunity against SARS‐2 typically decays rapidly after recovery and vaccinations may need to be repeated annually. Vaccines should aim to train the immune system more efficiently than natural infection through use of suitable adjuvants. Thus, immunization, and not natural infection, may be critical for generating and maintaining immunity.

Helper T‐cell epitopes should also be taken into account during vaccine development to generate immunological memory and reproducibility of immune responses. In addition, CD8^+^ T‐cell epitopes are also recommended for synergistic and superior protective immune responses. Recently mapped in SARS‐1‐S and SARS‐2‐S, CD4^+^, and CD8^+^ T‐cell epitopes are nonconformational (Figure 5 and 6, and **Table** **2**). A distinct advantage in peptide vaccines is that we are able to incorporate mapped T‐cell epitopes from other proteins in combination with neutralizing B‐cell epitopes in single molecularly defined construct. Recently, potent T‐cell epitopes in humans^[^ ^201^, ^202^ ^]^ and in mice^[^ ^203^ ^]^ were identified. The most promising CD4^+^ T‐cell epitopes within S‐protein are S_2T(h)5_“DYNYKLPDDFTGCVIAWNS,” S2_T(h)6_“RVVVLSFELLHAP,” and S_T(h)25_“NYNYKYRYLRGKLRPF.” The most potent CD8^+^ T‐cell epitopes were S_T8_ “NYNYLYRY,” S_T9_ “VNFNFNGL,” and S_T10_ “CYGVSPTKL.” Other immunodominant T‐cell epitopes were recently mapped in SARS‐2, and they were similar to those identified previously in SARS‐1. Further, HLA‐I A*02:01 restricted epitopes showed superior protection against SARS‐1 in lethal infection challenge using transgenic mice. HLA‐I A*02:01 is one of the most common human MHC‐I allele. Among these CTL‐epitopes, the most potent were S2_T8_^269^YLQPRTFLL^277^, **S_T3_** ^363^VADYSVLYNS^372^, **S_T2_** ^958^ALNTLVKQL^966^, S2_T10_^976^VLNDILSRL^984^, S2_T18_^1000^RLQSLQTYV^1008^, and **S_T1_** ^1192^NLNESLIDL^1200^ from SARS‐2‐S, Orf_1_2_T1_^3183^FLLNKEMYL^3191^, and Orf_1_2_T2_^1637^TTDPSFLGRY^1646^ from SARS‐2‐ORF‐1, and N2_T3_^322^MEVTPSGTWL^331^ and N_T1_^246^TVTKKSAAEA^255^ from SARS‐2‐N. N‐protein, ORF‐1, and S‐protein‐based T‐cell epitopes were found to induce IFN‐*γ* production from T‐cells of COVID‐19 recovered patients in vitro via Elispot stimulation. In these experiments, stimulation of CD^4+^ T‐cells by the helper T‐cell epitopes, for example, N2_T(h)1_^81^DDQIGYYRRATRRIR^95^ produced IFN‐*γ*. CD8^+^ T‐cells also produced IFN‐*γ* via stimulation with T‐cell epitopes, for example, N2_T3_^322^MEVTPSGTWL^331^.^[^ ^204^ ^]^ Interestingly, CD8^+^ T‐cell epitopes were able to protect 80–100% of either humanized HLA transgenic mice or BALB/c mice against lethal infection against SARS‐1. Since these epitopes are immunodominant in both SARS infections, it is highly likely that they play a similar protective role. Therefore, combining these T‐cell epitopes to support the principal protective humoral response may provide synergistic results and significantly improve host protection.

**Table 2:** Peptide‐based epitope mapping and peptide vaccine development against SARS‐1 and SARS‐2

Antigen–adjuvant	Animal modelDelivery route(D, day)	Efficacy and safety	Ref.
SARS‐1 B‐ & T‐cell epitopes			
HLA‐A2.1 restricted CD8^+^ T‐cell epitopes S_T1_^1174^NLNESLIDL^1182^ ^a)^ S_T2_^940^ALNTLVKQL^948^ S_T3_^349^VADYSVLYNS^358^ S_T4_^613^DQLTPAWRI^621^ N_T1_^246^TVTKKSAAEA^255^ M_T1_^143^IIRGHLRMAG^152^	HLA‐A2.1/H2‐D^b)^ hMHC‐I transfected C57BL/6 mice (*n =* 5) HLA‐A2 is highly expressed in Asians, Africans and Caucasians	CD4^+^ T‐cell epitopes were screened by measuring peptides’ (9‐ to 10‐mer) ability to stimulate IFN‐*γ* production and bind to HLA2.1‐MHC‐I of DCs and protect mice against lethal infection challenge. CD4^+^ T‐cell epitopes: S**_T1_** and S**_T2_** were potent inducers of IFN‐ *γ* and strong binders to MHC‐1, while S**_T3_** & S**_T4_** (located in the RBD), as well as **M_T1_** and **N_T1_** epitopes stimulated IFN‐*γ* production from convalescent patient T‐cells and mouse CD4^+^ T‐cells, but they were mild binders to HLA2.1 MHC‐I. S**_T1_** was the strongest binder and inducer of IFN‐*γ* production. S**_T1_**, S**_T2_,** S**_T3_**, and **N_T1_** epitopes are identical in SARS‐2, while S**_T4_** and **M_T1_** each differ by a single residue (I621V) and (M150I) from their SARS‐2 sequence.	^[^ ^211^ ^]^
HLA‐restricted memory T‐cell epitopes M_T(hC)2_^147^HLRMAGHSL^155^ N_T2_^266^TKQYNVTQAF^275^ N_T(h)3_^101^MKDLSPRWYFYYLGT^115^ S_T(h)5_^516^STDLIKNQCVNFNFN^530^ S_T(h)6_^541^SSKRFQPFQQFGRDV^555^ S_T(h)7_^1081^GTSWFITQRNFFSPQ^1095^	N‐ and M‐protein T‐cell epitopes were identified in three convalescent patients (HLA‐I A*2402, A*0206, A1101, A3303, A*0201, B*1502, B*1525, B*5502, B*5801, B*4001, C*0801, C*0403, C*0302, C*303 & C*1501), 9–11 years after SARS‐1 infection. The **M_T(h)2_** epitope was recognized as CD8^+^ T‐cell epitope in two patients and N**_T(h)3_** was recognized as CD4^+^ T‐cell epitope by two patients. N**_T2_** and M**_T(h)2_** epitopes resulted in a proliferation of IFN‐*γ* in vitro in 13% and 33% of CD8^+^ T‐cells, respectively. S**_T(h)6_**, S**_T(h)5_**, and S**_T(h)6_** epitopes induced CD4^+^ T‐cell IFN‐*γ* production from a single patient only. Each SARS‐1‐S epitope had three different residues from the SARS‐2 sequence, while M**_T(h)2_**, N**_T(h)3_**, and N**_T2_** epitopes had a single different residue (M150I), (D103E) and (Q268A), respectively. Two epitopes (S**_T(h)5_** and S**_T(h)6_**) were located in the RBD.		^[^ ^120^ ^]^
IV‐injected DCs pulsed with S_T8_, S_T9_, or both Recombinant Vaccinia virus (rVV)‐encoding S_T8_, S_T9_, or both S_T8_^436^NYNYKYRY^443^ (K440L) S_T9_^525^VNFNFNGL^532^ (100%)	C57BL/6J (*n =* 8–16) DCs: 10^6^ cells IV (D 0) rVVs 2 × 10^6^ PFU IN (D 6) SARS‐1: (MA15) 5 × 10^4^ PFU, (IN), C (42)^b)^	Long‐lived memory CD8^+^ T‐cells outlast B‐cell humoral responses by years. The IV administration of peptide pulsed DCs and IN administration of cytotoxic T‐cell epitope coated‐rVVs resulted in accumulation of antigen‐specific CD8^+^ T‐cells in lung fluid, in absence of SARS‐1‐specific CD4^+^ T‐cells or B‐cells. S_T_ **_9_** stimulated CD8^+^ T‐cell responses higher than those of S_T_ **_8_**, but both significantly stimulated CD8^+^ T‐cells and helped in their maturation to memory and poly‐functional phenotypes in vivo in mice. After lethal challenge, T‐cells produced IFN‐*γ*, IL‐2, and TNF‐a, and viral titers were reduced to 10^4.5^ and 10^2^ for S_T_ **_8_** and S_T_ **_9_** groups compared to the control (PBS) group (10^6^ PFU/g). Immunization with S_T_ **_8_**, S_T_ **_9_**, or both resulted in 60%, 80% and 90% survival rates in mice, respectively, compared to no survivors from the control group.	^[^ ^129^ ^]^
Early clodronate or Poly I:C [5 µg] Post‐challenge T‐cell‐specific responses S_T10_^366^CYGVSATKL^374^ (A370P) S_T11_^521^KNQCVNFNF^529^ (LS) S_T12_^1061^PAICHEGKAY^1071^ (E1067D, Y1071H) N_T(h)4_^353^ LNKHIDAYKTFPPTEPKK^370^ (100%)	BALB/c mice (*n =* 3–4) H2^d^ HLA SARS‐1 (MA15) IN 3 × 10^4^ PFU	The early macrophage depletion group showed no histological evidence of immunopathology in mouse lungs, compared to progressive interstitial pneumonia and diffuse alveolar damage in the control (PBS) group. Early depletion of alveolar macrophages using clodronate or maturation of DCs using TLR3 ligand poly I:C resulted in 100% survival of mice after lethal challenge. In contrast, stimulation with lipopolysaccharide (LPS) or late clodronate‐induced macrophage depletion resulted in lower survival (0% and 25%, respectively); no control mice survived. Antigen‐specific CD4^+^ and CD8^+^ T‐cell stimulation showed expanded populations of IFN‐*γ*‐producing T‐cells and antigen‐specific populations. Activated CD8^+^ T‐cells increased 4‐12‐fold with S_T_ **_10_**, CD4^+^ T‐cells increased 10‐fold with **N**_T_ **_(h)4_** epitope stimulation and 20‐fold by poly I:C. Passive transfer of mature activated DCs to naïve mice also resulted in 100% survival (similar to poly I:C and macrophage early depletion groups), and reduction in viral titers from 10^5–8^ to 10^2^ in the lungs, compared to the control group (no survivors). However, nAb titers and subclass were not evaluated.	^[^ ^64^ ^]^
S_B13_^471^ALNCYWPLNDYGFYTTTGIGYQPYRVVVLSFEL^503^ S_B14_^604^ TDVSTAIHADQLTPAWRIYSTGC ^625^ S_B15_^1146^ EIDRLNEVAKNLNESLIDLQELGKYEQYC^1191^ S_B16_^597^ LYQDVNCTDVSTAIHADQLTPAWRIYSTGC ^625^ [0.5 mg/kg]	Rhesus macaque monkeys (*n =* 6) IM (D 0, 14, 28, 42) SARS‐1 challenge 10^6^ TCID_50_	**S_B13‐15_** produced nAbs with high binding affinity (2–5 nm), while **S_B16_** produced Abs that enhanced viral entry into VERO E6 cells, even though the **S_B16_** sequence is mostly shared with **S_B15_**. The underlined sequences were recognized by IgG1 or IgG2a‐b mAbs from convalescent patient sera. **S_B14_** had the highest serological reactivity (33%) among neutralizing epitopes. A construct was prepared with four epitope copies in a dendrimer design via cysteine thiol‐acetamido linker. The vaccine combining **S_B13‐15_** induced IgG titers and reduced viral burden from 1.4 × 10^5^ to 7 × 10^3^ and the number of infected lung epithelia and macrophages were reduced by half, compared to the control group. **S_B13‐16_** mixture performed worse than **S_B13‐15_** due to viral activity enhancement of **S_B16_**; **S_B16_** alone performed worse than the negative control group. Lung pathology was significantly better with **S_B13‐15_** upon challenge, indicating infection control; alveolar damage with other vaccine groups was severe.	^[^ ^163^ ^]^
Orf_1B1_^1065^SDDYIKLNGPLTVG^1078^ (K1071A & T1076K) S_B17_^323^CPFGEVFNATKF^334^ (K333R) S_B18_^467^CTPPALNCYWPLND ^480^ (LS) S_B19_^545^FQPFQQFGRDVSDF^558^ (LS) S_B20_^651^PIGAGICASYHT^662^ (LS) S_B21_^663^VSLLRSTSQKSI^674^ **(LS)** (RRAR inserted at this cleavage site) S_B22_^695^AIPTNFSISITTEV^708^ **(LS)** M_B3_^165^KEITVATSRTLS^176^ (100%) M_B4_^5^GTITVEELKQLL^16^ (Q14K)	The structural (S, E, M, and N) proteins were synthesized as a series of 4942 10‐mer short overlapping peptide sequences. The peptides were tested for their potential recognition by neutralizing sera from four convalescent patients; healthy, unexposed patients; or by deceased patient sera. The Abs recognized by convalescent patient sera were potentially neutralizing and therapeutic; however, only simple linear epitopes can be recognized by this method. Other S‐, E‐, and N‐protein sequences were also found, but their protective efficacy is doubtful since they were either not found in three or more of the four sera (immune‐subdominant), or were not directed against M‐ or S‐proteins, as the other proteins are not as exposed on the virus surface. About 90% of produced Abs in convalescent SARS patients target the S‐proteins.		^[^ ^212^ ^]^
S_B22_^460^FSPDGKPATPPALNAYW^476^ (neutralizing) S_B23_^609^AIHADQLTPAWR^628^ (nonneutralizing) S_B24_^460^FSPDGKPCTPPALNCYWPLNDYGFYTTTGIGYQ^492^	High‐affinity neutralizing & nonneutralizing Abs were produced by hybridoma cell fusion to the antigen. The neutralizing Abs in general were found to bind to the RBD region. The most potent Ab in this library (G18) was found to bind to linear S_B22_ epitope and RBD with high affinity (1.78 nm), while nonneutralizing G8 was found to bind to a distant RBD epitope, S_B23_, with very high affinity (0.83 nm). Other highly potent nAbs were conformational and bound to the same region rich in critical binding residues, e.g., nAbs 80R and m396 bind to the 469–492 region of the RBD of S_B24_ with high binding affinities of 1.59 nm and 4.6 pm, respectively.^[^ ^148^, ^213^ ^]^		^[^ ^214^ ^]^
S_T(h)25_^435^NYNYKYRYLRGKLRPF^451^ S_T(h)26_^528^NFNGLTGTGVLTPSSKRF^545^ S_T(h)27_^633^AGCLIGAEHVDTSYECDI^650^ S_T(h)28_^649^DIPIGAGICASYHTVSLL^666^ S_T(h)29_^1082^SWFITQRNFFSPQII^1097^ Orf_3T(h)1_^36^PLQASLPFGWLVIGV^50^ M_T(h)5_^146^GHLRMAGHSLGRCDI^160^ N_T(h)5_^211^GETALALLLLDRLNQ^255^	The entire proteome of SARS‐1 was mapped for T‐cell epitopes by Elispot‐IFN‐g. Half of convalescent patients (*n =* 128) developed T‐cell responses. S‐protein was immunodominant as it covered most patients’ positive responses. 94% of recovered patients developed IgG titers against SARS‐1, and 91% of nAbs were directed against SARS‐1‐RBD with titers exceeding the 90% inhibitory concentration. This provides support for the theory that humoral responses are the more protective responses and correlates with infection survival estimates. nAb titers also correlated with T‐cell responses; however, T‐cell responses were not a prerequisite for recovery and seemed to develop slowly, which could be attributed to the inhibition of type‐1 INFs. In fatal SARS‐1 infection, serum levels of IL‐8, IL‐4, and IL‐5 were significantly higher than recovered patients’ levels (50, 3.4, and 0.64 pg mL^−1^, respectively). **S_T(h)25_** epitope was highly recognized by T‐cells (22 of 64 patients), followed by **S_T(h)27_** (17 patients), and **S_T(h)26_**, **S_T(h)28_**, and **S_T(h)29_** (7 patients). The ORF3 sequence was the only peptide recognized by T‐cells more than S‐protein peptides (24/64). **S_T(h)25_**, as well as ORF3, were CD8^+^ epitopes. IL‐12 (20‐fold), IFN‐*γ* (10‐fold), IL‐10 (10‐fold), IL‐6 (30‐fold), and IL‐8 (3‐fold) were elevated compared to normal levels: 3, 3, 1, 2, and 30 pg/mL, respectively, in serum seven days post‐onset.		^[^ ^189^ ^]^
M_B6_^1^MADNGTITVEELKQLLEQWNLVI^23^ M_B7_^132^LMESELVIGAVIIRGHLRMAGHPLGRCDIK^160^	Convalescent sera from 40 patients were mapped for immunodominant epitopes against SARS‐1 M‐protein. *C*‐terminal epitope (M_B7_) was more dominant and had higher titers against it in the sera. The most reactive epitopes of M_B6–7_ are listed. There are three transmembrane regions in the M‐protein sequence, starting from residue 15 and ending with 99. Therefore, Ab reactivity increased with the exposed portion of the protein. No in vitro neutralization or challenge studies were conducted.		^[^ ^215^ ^]^
**SARS‐2 mapped epitopes**			
**SARS‐2‐S B‐cell epitopes [25 ug dose]** S2_B1_^330^PNITNLCPFGEVFNATRFAS^349^ S2_B2_^370^STFKCYGVSPTKLNDLCFTN^395^ S2_B3_^450^NYLYRLFRKSNLKPFERDIS^469^ S2_B4_^480^CNGVEGFNCYFPLQSTGFQP^499^ **SARS‐2‐S T‐cell epitopes** S2_T(h)5_^420^DYNYKLPDDFTGCVIAWNS^439^ S2_T(h)6_^510^RVVVLSFELLHAP^521^	BALB/c mice (*n =* 5) S2_B2_, S2_B3_, or S2‐RBD were conjugated to KLH and mixed with alum SC (D 0, 14)	Epitopes from SARS‐2 N‐ and S‐proteins were mapped using 39 convalescent patients’ sera. Nine S‐protein immunodominant epitopes were identified (four epitopes in the RBD), compared to healthy volunteer sera. Two epitopes (S2B2, S2B3) were synthesized and tested as vaccine antigen in mice. They produced nAbs that had neutralizing activity comparable to the anti‐RBD Abs used as positive control. S2B2 and S2B3 contain also more likely T‐cell epitopes as shown by Elispot analysis, but S2_T(h)5_ and S2_T(h)6_ (S2_T(h)6_ > S2_T(h)5_) were potent in inducing IFN‐*γ* production from T‐cells.	^[^^103^, ^204^^]^
**SARS‐2 CD4^+^ T‐cell epitopes** N2_T(h)1_^81^DDQIGYYRRATRRIR ^95^ N2_T(h)2_^266^KAYNVTQAFGRRGPE^280^ N2_T(hc)3_^321^ **GMEVTPSGTWL**TYIGAIKLD^340^ **SARS‐2 CD8^+^ T‐cell** Within N2T(hc)3 MEVTPSGTWL	COVID‐19 convalescent patients (*n =* 37), Unexposed patients (*n =* 37), and SARS‐patients (*n =* 24)	Le Bert et al. explored virus‐specific T‐cell immunity against SARS‐2 epitopes from ORF‐1‐derived NSPs (esp. NSP‐7 and ‐13) and N‐protein via T‐cell Elispot. The specific T‐cell responses against ORF‐1 NSPs did not differentiate between unexposed patients and COVID‐19 recovered patients. In contrast, N‐protein, which is highly similar to SARS‐1‐N, matured T‐cells into CD4^+^, via N2_T (h)1_ (19% maturation) and N2_T(h)2_ (2% maturation), and CD8^+^ T‐cells, via N2T_3_ (2% maturation of the total population tested). N‐protein epitopes should, therefore, be included in peptide vaccines.	^[^ ^134^ ^]^
S2_B5_^655^HVNNSYECDIPIGAGICA^672^ S2_B6_^787^QIYKTPPIKDFGGFNFSQILPDPSKPSKPSKRSFIEDLL^822^	COVID‐19 convalescent patients (*n =* 12)	Linear neutralizing epitopes were mapped in SARS‐2 convalescent patients’ plasma. The epitopes are located at cleavage sites S2B5 at S1/S2, and S2B6 at S2’. The inhibition capacity of recovered patient Abs against S‐protein was tested via co‐incubation and gel electrophoresis.	^[^ ^216^ ^]^
**B‐cell epitopes** **S2_B7_**^451^YLYRLFRKSNLKPFERDIST^470^ **S2_B8_**^491^PLQSYGFQPTNGVGYQPYRV^510^ **S2_B9_**^391^CFTNVYADSFVIRGDEVRQI^410^ **S2_B10_**^331^NITNLCPFGEVFNATRFASV^350^ **S2_B11_**^341^FNATRFASVYAWNRKRISN^360^ **S2_B12_**^441^LDSKVGGNYNYLYRLFRKSN^460^	COVID‐19 convalescent patients sera (*n =* 7) BALB/c Mice (*n =* 4) Cysteinylated peptides were conjugated to diphtheria toxoid (DT) and 25 µg were mixed with Alum IM (D 0, 21, 28)	Seven RBD‐derived epitopes were mapped in convalescent patients’ sera by screening 20‐mer overlapping peptides. The peptides were conjugated to diphtheria toxoid (DT). DT‐peptides immune sera were tested for ACE‐2/RBD binding inhibition using competitive ELISA technique. While these sera triggered modest inhibition <30%, at 1/4 serum dilution. However, combining S2_B7_ and S2_B8_ immune sera resulted in significant inhibition ranging from 75% at 1/4 serum dilution down to an inhibition of 55% at 1/32 serum dilution. The combination of S2_B7_ and S2_B9_ was less effective than above. The peptides were not the most immunodominant epitopes in convalescent patient sera among RBD‐derived epitopes.	^[^ ^217^ ^]^
**N2_T4_** RTATKAYVN **N2_T5_** IIWVATEGA **N2_T6_** NTASWFTALT **S2_T7_** SIIAYTMSL **S2_T8_**^269^YLQPRTFLL^277^ **S2_T9_** RVVVLSFEL	N‐protein CD8^+^ T‐cell epitopes were identified in three convalescent patients’ PBMCs with different HLA‐I A alleles: A*02:06, A*24:02, A*02:01, A* 03:01, and A*24:07. Allele A*02:01 patient, which is one of the most common MHC‐I alleles, recognized N2T_5_. Other S‐ and N‐proteins epitopes were derived computationally with high allele‐population coverage, e.g., N2T_6_, S2T_7‐9_. S2T_8_ was confirmed in separate studies by flow cytometry in convalescent patients PBMCs.^[^ ^218^ ^]^		^[^ ^201^ ^]^
**Within S2_T8_**^269^YLQPRTFLL^277^ **S2_T10_**^976^VLNDILSRL^984^ **Orf_1_2_T1_**^3183^FLLNKEMYL^3191^	S‐protein derived A*02:01 allele restricted CD8^+^ T‐cell epitopes were identified in convalescent patient PBMCs, thus, priming host with these epitopes could help develop protective immune responses against infection. S2T_8_, S2T_10_, and Orf_1_2T_1_ stimulated IFN‐*γ* proliferation in PBMCs.		^[^ ^218^ ^]^
**Orf_1_2_T2_** TTDPSFLGRY **Orf_1_2_T3_** VYIGDPAQL **Orf_8_2_T1_** SKWYIRVGARKSAPL **S2_T11_** LTDEMIAQY **S2_T12_** QYIKWPWYI **S2_T(h)13_** ITRFQTLLALHRSYL **M2_T1_** NRFLYIIKL **M2_T(h)2_** LSYYKLGASQRVAGD **N2_T7_** KTFPPTEPKK **N2_T8_** ATEGALNTPK **N2_T9_** MEVTPSGTWL **N2_T(h)13_** KDGIIWVATEGALNT **N2_T(h)14_** GTWLTYTGAIKLDDK **N2_T(h)15_** RWYFYYLGTGPEAGL	PBMCs from 180 convalescent patients’ vs uninfected controls were used to examine CD4^+^ and CD8^+^ T‐cell epitopes throughout all of SARS‐CoV‐2 ORFs. The examination spanned common ten HLA‐1 alleles and six common HLA‐2 DR alleles. HLA‐1 alleles in CD8^+^ T‐cells A*01 (80%), A*01 (50%), A*03 (64%), A*11 (82%), A*24 (70%), B*40 (75%) and C*07 (55%) were stimulated (%) for IFN‐*γ* production via epitopes Orf_1_2T_2,_ S2T_11_, N2T_7,_ N2T_8,_ Orf_1_2T_3,_ N2T_9,_ and M2T1, respectively. Similarly, HLA‐2 DR alleles in CD4^+^ T‐cells were highly stimulated 91%, 77%,73%, 55%, 95%, and 68%, by N2T_(h)13,_ N2T_(h)14,_ N2T_(h)15,_ S2T_(h)13,_ M2T_(h)2_ epitopes, respectively. Several of these epitopes were found to stimulate nonexposed PBMC T–cells in milder manner compared to convalescent PBMC samples, e.g., N2T_(h)13_ (44%) due to sequence similarity with common cold corona viruses. In terms of T‐cell stimulation by structural and nonstructural whole proteins, HLA‐1 overall stimulation was in the following rank order S‐protein > M‐protein > Orf3, while HLA‐2 DR overall stimulation rank order was M‐protein >ORF8> E‐protein>N‐protein.		^[^ ^219^ ^]^
**Orf_1_2_T(h)4_**^3326^NHNFLVQAGNVQLRV **Orf_1_2_T(h)5_**^531^SPLYAFASEAARVVR **S2_T(h)14_**^816^SFIEDLLFNKVTLAD **S2_T(h)15_**^236^TRFQTLLALHRSYLT **S2_T(h)16_**^321^QPTESIVRFPNITNL **S2_T(h)17_**^316^SNFRVQPTESIVRFP	CD4+ T‐cell epitopes were screened in convalescent patients (*n =* 20) and in uninfected PBMCs samples. Four potent S‐protein derived CD4^+^ T‐cell epitopes were identified, that induced very high stimulation in several DRB and DQA HLA‐2 alleles, two of which were located in RBD sequence (S2T_(h)16–17_), while two others (S2T_(h)14–15_) were more potent and were located outside of RBD. Two more epitopes from Orf1 were also very potent in helper T‐cell stimulation. Overall helper T‐cell stimulation was higher for S‐protein than ORF1.		^[^ ^220^ ^]^
**Within S2_T8_**^269^YLQPRTFLL^277^ **S2_T18_**^1000^RLQSLQTYV^1008^	Convalescent patients (*n =* 17) and unexposed PBMCs were tested for CD8^+^ T‐cell stimulation with HLA‐1 A*02:01 restricted epitopes from SARS‐2‐S. S2T_8_ and S2T_18_ stimulated INF‐*γ* from CD8^+^ T‐cells of most patients’ PBMCs.		^[^ ^202^ ^]^
**BALB/c Epitopes** **N2_T(h)16_**^351^ ILLNKHIDAYKTFPP^365^ **S2_T19_**^268^GYLQPRTF^275^ **S2_T20_**^535^KNKCVNFNF^543^ **C57BL/6 Epitopes** **Orf_3_2_T(h)_**^266^EPIYDEPTTTTSVPL^280^ **M2_T3_**^174^RTLSYYKL^181^ **N2_T17_**^219^GFSALEPL^226^ **S2_T21_**^510^VVVLSFEL^517^ **S2_T22_**^538^CVNFNFNGL^546^ **S2_T23_**^820^DLLFNKVTL^828^	Epitopes were initially mapped in hACE2‐transgenic BALB/c and C57BL/6 mice to identify potent CD4^+^ T‐cell and CD8^+^ T‐cell epitopes and to examine their protective efficacy. A nanomolar dose of each peptide was adequate to stimulate IFN‐*γ* production in T‐cells within mice lungs and bronchoalveolar fluid. S2_T22_ and S2_T23_ were the most potent epitopes in hACE2‐C57BL/6 mice, while S2_T20_ was the most potent for hACE2‐BALB/c. Immunization of mice (hACE2‐BALB/c or hACE2‐C57BL/6) with N2_T(h)16_, S2_T19_, or S2_T20_ resulted in only partial protection in challenge experiments: 5‐ to 10‐fold PFU reductions in SARS‐2 virus were observed on the second day post‐challenge.		^[^ ^203^ ^]^
**S2_B13_**^63^TWFHAIHVSGTNGTKRFDNPV‐LP^85^ **S2_B14_**^92^FASTEKSNIIRGWIF^106^ **S2_B15_**^139^F LGVYYHKNNKSWM^153^ **S2_B16_**^406^EVRQIAPGQTGKIAD^420^ **S2_B17_**^439^NLDSKVGGNYNYLYR^454^ **S2_B18_**^455^FRKSNLKPFERDIS^469^ **S2_B19_**^475^GSTPCNGVEGFNCYFPLQSYGF‐QP^499^ **S2_B20_**^495^GFQPTNGVGYQPYR^509^ **S2_B21_**^793^PIKDFGGFNFSQILPDPSKP^812^ **S2_B22_**^909^GVTQNVLYENQKLI^923^ **S2_B23_**^1106^QRNFYEPQIITTDNT^1120^	Thirty‐three potential epitopes from various SARS‐2 proteins were used to immunize BALB/c mice and generate Ab titers. The generated Abs were evaluated for their neutralization efficacy using pseudovirion neutralization assays against the original SARS‐2 strain and the D614G mutant. The S2_B14_ and S2_B15_ epitopes neutralized both strains, while other epitopes were only effective against the D614 strain (S2_B13_). Antisera from NTD‐derived epitopes (S2_B13_‐S2_B15_) were highly neutralizing. Epitopes that were less protective against the D614 strain (S2_B19_‐S2_B29_) generated nAb titers of ≤ 30; moderately protective epitopes against both original and D614G strains (e.g., S2_B15_ and S2_B19_), and against the D614 strain (S2_B14_, and S2_B18_) generated nAb titers of 30–50; and epitopes that were highly protective against the G614 strain (S2_B13_, S2_B 14,_ S2_B21_‐S2_B23_) generated nAb titers of ≥ 50. CTD‐ and NTD‐derived epitopes (S2_B13_, S2_B23_) were more effective against D614G; however, RBD‐derived epitopes (S2_B16_, S2_B17_) were only slightly less effective. Notably, longer epitopes tend to adopt a conformation similar to those of the native protein, resulting in more potent nAbs.		^[^ ^221^ ^]^

^a)^ **S_T1_^1174^NLNESLIDL^1182^** is SARS‐1‐S derived T‐cell epitope, at position 1174 to 1182 of the full‐length Spike protein sequence. N and M, denote SARS‐1‐N and SARS‐1‐M proteins derived epitopes, while S2 denotes SARS‐2‐S derived epitopes, N2 and M2 denote SARS‐2‐N and SARS‐2‐M proteins derived epitopes, respectively. The B subscript denotes a B‐cell epitope, T subscript denotes T‐cell epitope, and T(h) subscript denotes a helper T‐cell epitope. SARS‐1‐derived epitopes with low sequence identity to SARS‐2 (LS) have more than 3 different residues.

^b)^ **(IN), C (42)** is challenge on day 42 from day zero (immunization), infection via intranasal route.

Ab, antibodies; C, challenge; D, day; DC, dendritic cells; HLA, human leukocyte antigen system; IM, intermuscular; IN, internasal; IP, intraperitoneal; KLH, Keyhole limpet hemocyanin; LPS, bacterial lipopolysaccharide, pro‐inflammatory Th‐2 adjuvant; MA15, a mouse adapted SARS‐1 strain; MHC, major histocompatibility complex; nAb, neutralizing antibody; PFU, plaque forming unit; SC, sub‐cutaneous; TCID, tissue culture infective dose 50%.

The neutralization efficacies of the currently approved vaccines dropped significantly when they were tested against recently emerged SARS‐2 variants. To date, mutations have been observed in viral structural and NSPs, including ORF‐1, S‐, E‐, and N‐proteins. Importantly, mutations in the RBD sequence have also been reported. SARS‐2‐S mutations have occurred in the P1 lineage (L18F, T20N, P26S, D138Y, R190S, K417T, E484K, N501Y, and D614G), the B.1.1.7 lineage (del69–70 HV, del144 Y, N501Y, A570D, D614G, P681H, T761I, S982A, and D1118H), and the B.1.351 lineage (K417N, E484K, N501Y, D614G, and A701V). All approved vaccines have efficacies of ≥85% against the original SARS‐2 virus strain. However, the efficacy of the ChAdOx‐1 nCoV‐19 (AstraZeneca/Oxford) vaccine dropped to 70% and 81% against the B.1.1.7 (T20I and N501Y) and non‐(B.1.1.7) lineages (“UK variants”), respectively.^[^ ^178^ ^]^ Similar reductions in efficacy were also found in the context of the South African variants, for example, B1.351 with a N501Y mutation.^[^ ^205^ ^]^ Further, the mRNA‐1273 vaccine (Moderna) was found to have a 5‐ to 10‐fold reduced efficacy against the B.1.351 variant in nonhuman primates and in sera isolated from immunized humans.^[^ ^206^ ^]^ However, it still provides sufficient protection against the B.1.1.7 lineage (based on pseudovirion nAb titers of >300).^[^ ^207^ ^]^ The P1 variant, which is common in Japan and Brazil, possesses similar mutations (T20J and N501Y) to those reported to be highly resistant to vaccination with formulations utilizing the original SARS‐2‐S sequence.

The main reason for reduced vaccine efficacy has been mutation at or near the SARS‐2‐S binding motif. However, the *N*‐terminus domain (NTD) of the S‐protein has been shown to include potent neutralizing epitopes^[^ ^196^, ^208^ ^]^ and mutations in this region have also impaired vaccine efficacy. Immune sera against the original SARS‐2‐S were effective against the D614G mutation (nAb titers of 1800) in nonhuman primates^[^ ^206^ ^]^ suggesting that this mutation is less detrimental to S‐protein‐based vaccine efficacy. However, it is important to note that these outcomes are mainly resultant from in vitro neutralization‐based assays. Contrary to humoral (antibody‐based) responses, convalescent patient T‐cells continue to recognize mutant strains,^[^ ^209^ ^]^ as most T‐cell epitopes have remained conserved between variants. Combining cellular and humoral responses could protect vaccine efficacy against future emerging variants, even if the main neutralizing epitopes are altered. Therefore, vaccine efficacy against mutants cannot be determined by simple serum‐based naturalization assays; instead, infection challenge studies in mice and nonhuman primates are required, and, more importantly, longitudinal studies on post‐vaccinated human subjects.^[^ ^178^, ^210^ ^]^

### 4.3 Vaccine Antigen and ACE2 Binding

Interestingly, one type of APCs: macrophages, express ACE2.^[^ ^222^, ^223^ ^]^ This not only implies that macrophages are vulnerable to infection in the lungs, but also that S‐protein and its RBD when used as vaccine antigen could preferentially bind to macrophage surface‐expressed ACE2. All subunit‐based vaccine formulations contain an adjuvant and B‐cell epitope/s (possibly in the form of whole protein) intended to target pathogen recognition receptors on APCs, for example, dendritic cells, and B‐cells, respectively. The binding of S‐protein or its antigenic fragments to macrophage‐ACE2 can be an alternative to antigen binding to B‐cell receptors for the production of Abs.^[^ ^115^, ^117^ ^]^ This leads to off‐target loss of antigen. Simultaneously, the effects from ACE2‐downregulation due to RBD‐ACE2 binding causes a switch toward inflammatory responses and delay in the development of adaptive immune responses due to the combined effects of antigen underdosing and ACE2‐downregulation.^[^ ^87^, ^154^ ^]^ DNA‐ and RNA‐based vaccines may initially circumvent this major drawback of off‐target dose wasting and preserve the S‐protein in prefusion conformation.^[^ ^168^ ^]^

However, after the translation and secretion of S‐protein, nonspecific ACE2 binding could occur during antigen presentation. The gradual release of RNA‐translated S‐protein demonstrated much milder immunopathology compared to protein‐based vaccine antigens and developed the required noninflammatory adaptive immune response necessary for protection.^[^ ^166^ ^]^ This effect was also observed when low‐dose S‐protein was utilized as vaccine antigen. However, relatively low nAb titers were observed and immunopathology was not completely prevented.^[^ ^154^ ^]^ While the low efficacy of DNA and RNA vaccines results from limited transfection efficacy, the use of nontoxic and safe transfection agents, for example, lipoplexes, reduces the chance of side‐effects. In contrast, more effective nonreplicating viral vectors induce fever and have safety concerns.^[^ ^224^ ^]^ These critical drawbacks can be completely avoided by employing peptide‐based subunit vaccines combined with suitable adjuvants to stimulate a specific effective immune response.

### 4.4 Considerations Regarding Antibody Subclass and Proper Adjuvant Selection

Antibody subclass has an important role in protection against SARS infections, as each subclass varies in its involvement in inducing pro‐inflammatory responses, recruitment of inflammatory cells, and in opsonization by antibody‐dependent cytotoxic effects. Danger signals recognized by APCs drive interleukin production. The interleukin profile then drives immunoglobulin class‐switching, for example, IL‐10 drives immunoglobulin class‐switching to IgG4. Hence, proper adjuvant choice is one of the most crucial steps in vaccine development.^[^ ^225^, ^226^ ^]^ For example, as an adjuvant, alum normally generates IgG1 as the dominant subclass with very minor IgG2_a,c_ levels, while Th1‐supporting adjuvants, poly I:C and CPG ODN, generate IgG2_a,c_ as the dominant subclass.^[^ ^227^, ^228^ ^]^ Recently, SARS‐2‐S protein was tested for its efficacy in generating nAbs against SARS‐2 in combination with adjuvants, such as AS03, AS37, Alum, and Alum‐CPG1018. AS03 followed by Alum‐CPG1018 outperformed the other adjuvants tested in terms of generating stable, high levels of nAb titers > 10^3^ (*n* = 5–6 mice per group).^[^ ^229^ ^]^

Soluble and transmembrane protein antigens recognized by B‐cells trigger the production of IgG1 and IgG3, while IgG4 and IgE production results from repeated exposure to allergens, which provokes inflammatory allergenic or anti‐inflammatory responses, respectively.^[^ ^230^, ^231^ ^]^ For example, carbohydrate antigens almost exclusively result in IgG2 responses.^[^ ^232^, ^233^, ^234^, ^235^, ^236^ ^]^ The natural ratio of IgG subtypes IgG1/IgG2 is 2:1, unless a strong, specific, class‐switching adjuvant is employed. In early viral infection stages, the levels of IgG3 are initially elevated and class‐switching follows to the less inflammatory IgG1 subclass.^[^ ^233^ ^]^ APCs bear FC*γ*R and FcRn receptors, which amplify signaling and interleukin production, including pro‐inflammatory cytokines (Figure 4). Activating FC*γ*R receptors triggers inflammatory responses, including the release of chemo‐attractants, cytotoxic substances from immune effector cells, and pro‐inflammatory mediators, thus recruiting inflammatory immune cells. IgG3 and IgG1 bind very strongly to most FC*γ*R receptors, whereas IgG2 binds very weakly to all of them except FC*γ*R II_a_.^[^ ^237^ ^]^ Since IgG2 binds weakly to FC*γ*R receptors, triggering IgG2 mediated responses should reduce undesired inflammatory responses. The strong binding of IgG1 and IgG3 to FC*γ*R‐III_b_ present on neutrophils could be one of the leading causes of lung immunopathology in early infection. In contrast, IgG2 does not bind to FC*γ*R‐III_b_. This may explain the safe and protective Th1‐biased humoral responses achieved by combining SARS‐1‐S with TLR3 adjuvant (poly I:C) in mice facing infection challenge, although only total neutralizing IgG titers were evaluated in the study.^[^ ^154^ ^]^ Similar IgG2 mediated protective responses with minimal immunopathology were induced by influenza vaccine in mice.^[^ ^153^, ^154^, ^238^ ^]^

IgG1 and IgG2 also bind to the FcRn receptor, which is widely expressed on epithelia, including respiratory epithelia and myeloid cells, thus complementing the role of IgA in mucosal immunity. Another inflammatory mechanism—binding of IgG to C1q protein—is also triggered by complement activation. IgG2 is the weakest C1q binder, compared to IgG3 and IgG1.^[^ ^239^, ^240^ ^]^ In summary, IgG2 has prolonged circulation half‐life, contributes to mucosal immunity, and has reduced inflammatory effects, due to low C1q and FC*γ*R binding, but retains the protective functions of opsonization and neutralization of Abs.

Careful adjuvant selection is important in helping to develop adaptive immune responses; as engineering of the produced interleukin profile can directly drive maturation and recruitment of immune cells. APCs express several pathogen recognition receptors, including TLRs, for which MyD88 is an adaptor signaling protein and key for interleukin production. MyD88 signaling in APCs was found to be essential for protection against SARS‐1 infection in mice. In a recent study, MyD88 −/− C57BL/6 mice were more susceptible to infection by SARS‐1 MA15 strain (>90% of mice died), whereas all of the wild‐type black mice survived. In addition, CCR1 is the MCP‐1 cytokine receptor expressed in APCs, T‐cells and neutrophils. CCR1‐deficient mice exhibited exacerbated immunopathology, with only 60% survival.^[^ ^143^ ^]^ CCR1 triggers pro‐inflammatory responses in early infection; however, it also induces protective innate responses to some extent in early infection. In contrast to innate responses, early recruitment of professional DCs and the avoidance of pro‐inflammatory cell recruitment alleviated symptoms and generated a more protective adaptive immune response.^[^ ^118^ ^]^ Therefore, vaccine against SARS‐2 should preferably induce balanced Th1/Th2 or even biased Th1 and Th17 responses at the expense of Th2‐type responses. Therefore following Th1‐biased adjuvants have been suggested as promising immune stimulant for SARS vaccine formulations: a) IC30, cationic polypeptide with TLR9 agonist (Th1); b) GLA SE, a squalene‐based water‐in‐oil emulsion with TLR4 lipid agonist (Th1); c) CAF01, a cationic liposome with mincle receptor agonist (Th1/Th17); d) poly I:C, a double‐stranded RNA as a TLR3 agonist, (Th1/Th17); and d) MF59, a squalene‐based oil‐in‐water emulsion (Th1/Th2 balanced). Adjuvancy power, expressed as total IgG humoral responses, varies among these adjuvants. When hemagglutinin was applied as model antigen the level of Ab titers decreased in following order MF59 > GLA SE > CAF01 > alum, after two subcutaneous immunizations in mice.^[^ ^241^ ^]^ The most common adjuvant used in vaccine formulations worldwide, alum, was not only the weakest among them but it also stimulates Th2 rather than desired Th1 immune responses.

### 4.5 In Vitro Efficacy Screening Considerations

Several in vitro tests were developed to investigate potential protection against SARS infections via humoral responses. Pseudovirion cell entry inhibition, antigen‐Ab binding affinity, for example, by surface plasmon resonance, and competitive ELISA tests have all been used to assess specific inhibitory activities of produced Abs against viral fusion proteins. Binding affinity tests assess when generated Abs have a strong enough binding affinity to S‐protein or RBD to block S‐protein binding to ACE2. In competitive ELISA tests, plates are either coated with ACE2 or RBD followed by immune sera, then the other protein (RBD or ACE2, respectively) is added to explore how binding was affected or inhibited by the sera.^[^ ^242^ ^]^

Pseudovirion cell entry inhibition provides the closest simulation of cell infection. ACE2‐transfected HEK cells are often employed with a mixture of animal sera at different dilutions and pseudo‐virus particles (lentivirus) bearing S‐protein added to ACE2 cells. This allows for exploration of binding inhibition between S‐protein and ACE2 in an environment that mimics in vivo conditions. Recently, a strong correlation was demonstrated between nAb titers and the pseudovirion entry test (*R* ^2^ = 0.83);^[^ ^139^, ^157^ ^]^ this supports the suitability of employing simple in vitro tests, such as competitive ELISA, in preclinical vaccine candidate screening in early development stages. However, these tests, including pseudovirion entry, often fail to assess the true protective efficacy of Abs with nonneutralizing protective mechanisms, for example, opsonizing Abs. For example, Abs generated against several conserved epitopes in the influenza fusion protein (hemagglutinin) were protective in vivo, but did not exhibit cell entry inhibition in vitro. Similarly, this assay failed to assess the neutralization of a protective Ab (7D10) against a conserved neutralizing epitope at the NTD of MERS‐CoV‐S. The test failed, as 7D10 binds to the NTD of MERS‐CoV‐S only when the latter is bound to DPP‐4 receptor and the epitope is accessible.^[^ ^197^ ^]^ In such cases, competitive ELISA tests are incompatible; however, pseudovirion cell entry test may assess efficacy depending on design.^[^ ^197^ ^]^ Several studies demonstrated the protective power of nonneutralizing Abs against lethal SARS and influenza infections, even though they failed to demonstrate protection via in vitro pseudovirion cell entry inhibition tests.^[^ ^153^, ^185^, ^186^, ^187^, ^238^, ^243^ ^]^ Therefore, depending on the mechanisms of protection offered by antigen‐specific Abs, the efficacy of in vitro assessment testing for evaluating protective role will vary.

## 5 Animal Models for Vaccines against SARS‐2: Efficacy and Safety Evaluation

A variety of distinct animal models have been used to study SARS infections. Models were selected based on their ability to mimic pathological symptoms and the lethality of human infection. A few studies employed ferrets as animal models for SARS‐1 infection challenges;^[^ ^96^, ^97^, ^244^ ^]^ however, these models are partially resistant to SARS‐2 infection.^[^ ^245^, ^246^ ^]^ For example, while ferrets are susceptible to infection, they require high SARS‐2 dose (10^5.5^ PFU) to enable quantification of viral RNA titers, and to exhibit histopathological symptoms, pneumonia, and lung lesions.^[^ ^246^ ^]^ Viral RNA titers peak 2–6 days post‐infection, then viral clearance occurs by day 14 post‐infection. Ferrets are also immune to reinfection. To date, only two SARS‐2 infection challenge studies have utilized ferrets for the purpose of examining vaccine efficacy.^[^ ^177^, ^247^ ^]^ In contrast, Syrian hamsters have been recently employed as a suitable infection challenge animal model for SARS‐2; this model is susceptible to infection using relatively low SARS‐2 dose (10^3.5^ PFU).^[^ ^248^, ^249^ ^]^ Importantly, viral RNA titers are detectable, days 2–7 post‐infection, in nasal turbinates, duodenum, feces, brain, and kidneys.^[^ ^249^ ^]^ Infected hamsters exhibit similar transmissibility and histopathology to infected patients, including pneumonia, lung lesions, and inflammatory cells infiltration.^[^ ^248^, ^249^ ^]^ The infection lasts for 14 days post‐infection, when infection is cleared, humoral immune responses protects the animals against reinfections. Hamsters have been recently used to evaluate vaccine efficacy.^[^ ^170^, ^174^, ^250^, ^251^ ^]^ Thus, hamster represents a promising highly susceptible infection challenge model for SARS‐2.

BALB/c mice are the most common models for SARS‐infection challenge, due to compatibility with pro‐inflammatory pathological responses in early stages of infection.^[^ ^203^, ^252^, ^253^, ^254^ ^]^ Upon SARS‐1 infection, mice produce a wave of pro‐inflammatory cytokines (TNF‐*α*, IL‐6) and chemokines (CXCL‐10, CCL2, CCL3, CCL5) by day 2 post‐infection. In response to chemotaxis, NK‐cells, immature macrophages, and DCs are recruited to the lungs. On day 7 post‐infection, a second wave of cytokines and chemokines are produced, in addition to IFN‐*γ*, IL‐2, IL‐5, and CXCL‐9. Similar acute lung injury with diffuse alveolar damage occurs due to immunopathology. However, SARS‐1 infection in BALB/c mice is not highly lethal, which reduces the applicability of these mice for demonstrating the protection efficacy of vaccine candidates.

Two approaches were adopted to develop more sensitive mouse models to mimic lethality and pathological symptoms. The first approach employed transgenic mice expressing human ACE2 (hACE2).^[^ ^255^ ^]^ Transgenic hACE2 mice were able to mimic the infectivity of SARS‐cell entry observed in primary hosts.^[^ ^35^, ^38^, ^256^, ^257^ ^]^ Infection of hACE2‐transgenic mice with TCID_50_ SARS‐2 doses of 3 × 10^4^ and 7 × 10^5^ resulted in 50% and 100% lethality, respectively, by fifth day post‐infection (*n =* 4–6). Thus feasible lethal infection challenge model was created.^[^ ^258^ ^]^ Alternatively, lungs of regular mice can also be transfected/transduced, with hACE2 via adenovirus serotype‐5 (Ad5) encoding hACE2, by intranasal administration, thus rendering them susceptible to SARS‐2 infection. Infection of the hACE2‐Ad5‐transduced BALB/c or C57BL/6 mice with 2.5 × 10^8^ PFU virus dose resulted in steady viral RNA lung titers of 10^10^ g^−1^, and PFU g^−1^of 10^5^, at fourth to eighth day post‐infection. Additionally, this transduced mouse model exhibited sensitivity to neutralization by nAbs, detectable lung histopathological damage by hematoxylin and eosin tissue staining, and significant increase in cytokine storm, that is, IL‐6, CXCL‐10, and IL‐28_a/b_, with infection progression and a decrease in body weight.^[^ ^253^ ^]^ The second approach involved developing a special SARS‐virus strain that was more suited for mouse lethal infection. MA15 SARS‐1 strain infected both black C57BL/6^[^ ^259^ ^]^ and BALB/c mice^[^ ^64^ ^]^ with SARS‐1. Recently, a similar approach was adopted for SARS‐2 infection in old and young BALB/c mice, on third day post‐infection with the N501Y mutant SARS‐2 strain, the infected mice had steady SARS‐2 viral RNA lung titers of 10^11^ g^−1^, detectable cytokine storm, a decrease in body weight with infection progression, and detectable lung histopathological damage via hematoxylin and eosin tissue staining.^[^ ^252^ ^]^ Therefore, similar pathology in animal models is important to allow for not only vaccine efficacy, but also safety.^[^ ^259^ ^]^ Ultimately, both of these approaches achieved the required degree of susceptibility to immunopathology and lethality in control mice, thus rendering them sensitive and suitable animal models for comparing the protective efficacy of vaccines while monitoring immunopathological adverse events, especially after infection challenge.

Rhesus macaque monkeys are a common final preclinical model with close physiological, histological, immunological, and immunopathological responses to human infection by SARS‐1 and SARS‐2.^[^ ^260^ ^]^ They possess highly homologous ACE2 to humans, which is expressed in similar tissues.^[^ ^257^, ^261^ ^]^ The rhesus monkey model was recently employed to provide proof‐of‐concept on the safety and efficacy of S‐protein‐encoded DNA‐loaded chimpanzee adenovirus (Chadox‐1) vector.^[^ ^168^ ^]^ These animal models are considered adequate for establishing preclinical efficacy and safety before proceeding to phase I human clinical trials.

## 6 Conclusions

Even after the current COVID‐19 pandemic disaster is curbed, spillover from viral reservoir animal hosts to humans could occur again. In order to be as prepared as possible, it is extremely important to progress extensive research in vaccine development, molecular pathology, and surrogate in vitro efficacy tests to fast‐track effective medical interventions.

It is highly likely—and possibly essential—that a peptide‐based subunit vaccine is produced against SARS‐2, as this type of vaccine has several advantages over protein and RNA‐based vaccines. Peptide‐based vaccine can a) incorporate epitopes from different proteins in one immunogenic construct, thus exceeding the efficacy of a single epitope, b) support the initiation of different protective immune mechanisms simultaneously, and c) avoid unnecessary immunopathological side effects. Moreover, peptide vaccines are easy to chemically synthesize, process and purify; are scalable for mass production to meet current global demand; are stable as dry, sterile powder; can be stored/transported without needing to be kept cold; and are devoid of biological contaminants. Furthermore, combining peptide vaccine with proper Th1 and Th17 interleukin‐inducing adjuvants is achievable to avoid hyperreactive Th‐2 responses that exacerbate immunopathology.

Peptide‐based vaccines can enclose opsonic or neutralizing epitopes, or both. Neutralizing B‐cell epitopes that contain several CBRs prevent mutant escape; as mutations of these moieties will also reduce virus infectivity. Convalescent patient sera, even with low nAb concentration, can neutralize the virus. Three neutralizing epitopes (pep1‐3) are suggested here as potential neutralizing peptide vaccine antigens. Moreover, the incorporation of cytotoxic T‐cell epitopes and B‐cell epitopes in the vaccine may provide synergistic protective effects.

In conclusion, peptide‐based vaccines against coronaviruses have been somewhat overlooked, but they hold great potential for delivering safe and protective immune responses against SARS‐2 infections.

## Conflict of Interest

The authors declare no conflict of interest.

## Supporting information

Supporting Information
