# Supplementary material for: Key Considerations for the Development of Safe and Effective SARS‐CoV‐2 Subunit Vaccine: A Peptide‐Based Vaccine Alternative
Source: Adv Sci (Weinh). 2021 Jun 27;8(16):2100985. doi: 10.1002/advs.202100985 (PMC8373118; doi:10.1002/advs.202100985)
Supplement: Supplementary file 1 — Supporting Information [file ADVS-8-2100985-s001.pdf]

© 2021 Wiley-VCH GmbH

ADVANCED SCIENCE
Open Access

Supporting Information

for *Adv. Sci.*, DOI: 10.1002/advs.202100985

Key considerations for the development of safe and effective SARS-CoV-2 subunit vaccine: A peptide-based vaccine alternative

Ahmed O. Shalash, Waleed M. Hussein, Mariusz Skwarczynski, and Istvan Toth**

Supporting Information

Key considerations for the development of safe and effective SARS-CoV-2 subunit vaccine: A peptide-based vaccine alternative

Ahmed O. Shalash, Waleed M. Hussein, Mariusz Skwarczynski, Istvan Toth**

The blood levels of cytokine storm and lymphocyte counts were compared between SARS-1 and SARS-2 infected patients with mild to severe illness during infection progress (Figure S1).

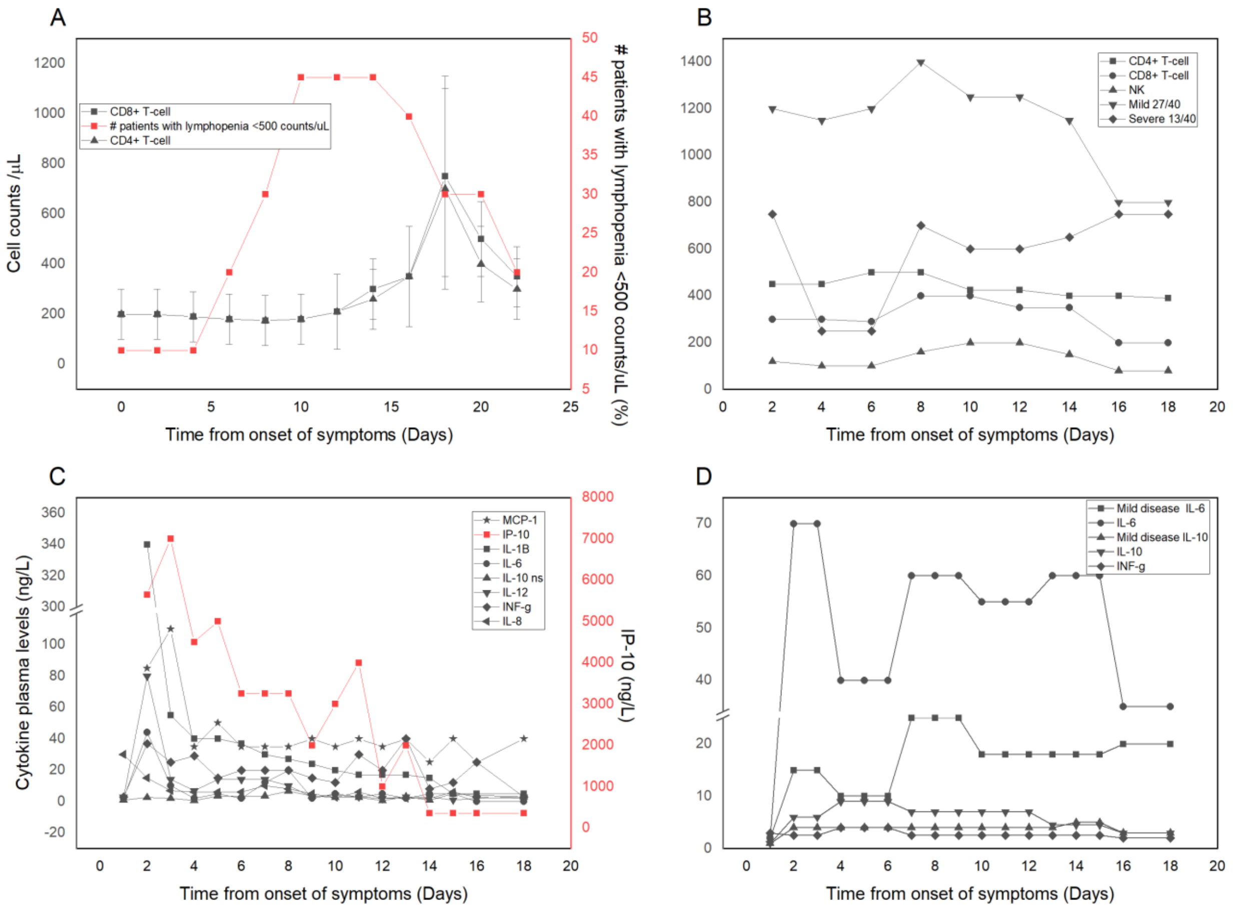

Supporting Figure S1. Cell count and cytokine response profiles in the plasma of SARS-2 and SARS-1 infected patients from the onset of symptoms. T-cell responses and lymphopenia in SARS patients (n=157) (A); T-cell and natural killer cell (NK) responses in COVID-19

patients (n=40), including $CD8^+$ T-cell counts in mild and severe cases (B); cytokine and interleukin profiles of SARS-patients (n=20) (C); and cytokine and interleukin profiles of COVID-19 patients showing mild and severe illness levels (n=40) (D). Adapted from [49, 57, 61].
